# Integrated genome-wide DNA methylation and transcriptomic analysis prioritizes *EPHA5* as a candidate non-invasive epigenetic biomarker for oral squamous cell carcinoma

**DOI:** 10.3389/fonc.2026.1932336

**Published:** 2026-10-02

**Authors:** Semra Demokan, Begüm Özemek, Seval Turna, Sevde Cömert, Cömert Şen, Kağan Avcı, Ahmet Özkan, Sena Şen, Mehveş Poda, Murat Ulusan, Uğur Sezerman, Gülsüm Ak, Canan Alatlı, Nejat Dalay

**Affiliations:** 1 Department of Basic Oncology, Oncology Institute, Istanbul University, Istanbul, Türkiye; 2 Department of Biostatistics and Medical Informatics, Faculty of Medicine, Acibadem Mehmet Ali Aydınlar (MAA) University, Istanbul, Türkiye; 3 Institute of Graduate Studies in Health Sciences, Istanbul University, Istanbul, Türkiye; 4 School of Health Services Vocational College, Pathology Laboratory Techniques Program, Bezmialem Vakif University, Istanbul, Türkiye; 5 Department of Otorhinolaryngology and Head and Neck Surgery, Istanbul Faculty of Medicine, Istanbul University, Istanbul, Türkiye; 6 Department of Genetics, Aziz Sancar Institute of Experimental Medicine, Istanbul University, Istanbul, Türkiye; 7 Department of Oral and Maxillofacial Surgery, Faculty of Dentistry, Istanbul University, Istanbul, Türkiye; 8 Department of Medical Pathology, Medical Faculty, Istanbul Health and Technology University, Istanbul, Türkiye

**Keywords:** *EPHA5*, epigenetic biomarker, genome-wide DNA methylation, liquid biopsy, oral premalignant lesion, oral squamous cell carcinoma, saliva, transcriptomics

## Abstract

**Purpose:**

Oral squamous cell carcinoma (OSCC) remains a major clinical challenge because of delayed diagnosis and the lack of validated non-invasive biomarkers for OSCC detection. Epigenetic alterations during oral carcinogenesis represent promising biomarker candidates. This study aimed to prioritize clinically relevant epigenetic biomarkers and validate *EPHA5* using an integrated genome-wide DNA methylation and transcriptomic approach.

**Experimental design:**

Genome-wide DNA methylation and transcriptomic profiling were performed to identify epigenetically deregulated genes in OSCC. Candidate genes were prioritized by integrating DNA methylation, transcriptomic data and biological relevance. *EPHA5* was subsequently selected for validation and evaluated by quantitative methylation-specific PCR (QMSP) and quantitative real-time PCR (QRT-PCR) in tissue, saliva and serum samples from independent cohorts comprising patients with OSCC, oral premalignant lesions (OPML) and healthy controls.

**Results:**

Genome-wide analysis identified 661 upregulated and 577 downregulated genes together with 2,431 differentially methylated regions corresponding to 1,776 genes. Integrated analysis generated a panel of 51 candidate genes, from which *EPHA5* was prioritized using integrated DNA methylation, gene expression, pathway enrichment and biological relevance. *EPHA5* promoter hypermethylation was detected in 66.7% of OSCC tumors, whereas concurrent promoter hypermethylation and reduced expression were observed in 28.6% of tumors. Methylation was also detected in 54.5% of OPML lesions and in the saliva of 47.6% of OSCC patients but was rarely detected in healthy controls. Tumor *EPHA5* methylation significantly discriminated OSCC from healthy controls (AUC = 0.780, p = 0.004; sensitivity, 71.4%; specificity, 75%). Salivary *EPHA5* methylation also significantly discriminated OSCC from healthy controls (AUC = 0.717, p = 0.025; sensitivity, 52.4%; specificity, 87.5%), whereas serum methylation showed no significant diagnostic utility (AUC = 0.571, p = 0.462). Discrimination between OSCC and OPML was limited and did not reach statistical significance (tumor AUC = 0.662, p = 0.068; salivary AUC = 0.600, p = 0.264).

**Conclusions:**

Frequent tumor hypermethylation together with detectable salivary methylation supports further evaluation of *EPHA5* as a candidate non-invasive epigenetic biomarker for OSCC. However, its moderate diagnostic performance, limited discrimination between OSCC and OPML, and lack of external validation indicate that larger prospective studies and evaluation within multimarker methylation panels are required before clinical implementation.

## 1 Introduction

Oral squamous cell carcinoma (OSCC) is one of the most common malignancies worldwide, ranking sixth among all cancers in global prevalence (1). It predominantly affects individuals over 50 years of age, particularly men and most frequently arises in the tongue, lips and floor of the mouth (2). The development of OSCC is often preceded by the malignant transformation of oral premalignant lesions (OPML), including oral leukoplakia (OL), oral lichen planus (OLP), erosive oral lichen planus (EOLP) and oral lichenoid dysplasia (OLD). Although all of these lesions possess malignant transformation potential, they differ in their clinicopathological characteristics and associated risks. OL is generally regarded as a higher-risk precursor, whereas OLP, EOLP and OLD belong to the spectrum of lichenoid oral potentially malignant disorders (3). Despite advances in diagnosis and treatment, OSCC remains a major global health burden, with 5-year survival rates ranging from 70-90% for early-stage disease but declining to 30-50% in patients with advanced-stage (4).

Although OSCC is fundamentally driven by genetic alterations, environmental and lifestyle factors play a pivotal role in its initiation and progression. Tobacco use, alcohol consumption, betel quid chewing, chronic inflammation and human papillomavirus infection are well-established risk factors, with tobacco and alcohol increasing the risk of OSCC by up to sixfold (5). Increasing evidence suggests that many of these environmental exposures exert their carcinogenic effects through epigenetic mechanisms, particularly DNA methylation. Aberrant epigenetic alterations have been implicated in tumor initiation and progression by deregulating gene expression and disrupting essential biological processes, including cell cycle regulation, DNA repair, cell adhesion, signal transduction, apoptosis and cellular differentiation (6).

Histopathological evaluation of tissue biopsy remains the gold standard for OSCC diagnosis. However, increasing attention has focused on the development of non-invasive diagnostic approaches, including liquid biopsy, optical imaging systems, oral brush cytology, microfluidic technologies, nanotechnology-based detection platforms and artificial intelligence-assisted image analysis (7). Among these approaches, liquid biopsy has emerged as a promising strategy because it enables the assessment of tumor-associated molecular alterations in easily accessible body fluids such as saliva and blood. These alterations include circulating tumor cells together with genetic and epigenetic abnormalities, particularly aberrant promoter DNA methylation (8). Previous studies have shown that aberrant promoter DNA methylation frequently occurs during oral carcinogenesis and may precede clinically detectable disease, making DNA methylation an attractive source of candidate biomarkers for disease detection and monitoring (9).

Although an increasing number of DNA methylation biomarkers have been proposed for OSCC, none has yet been translated into routine clinical practice for non-invasive detection. Therefore, the present study aimed to prioritize and independently validate candidate DNA methylation-based biomarkers for OSCC using an integrated genome-wide methylation and transcriptomic strategy. Candidate genes were prioritized by integrating genome-wide DNA methylation and transcriptomic data with pathway enrichment analysis, biological relevance and translational potential rather than statistical ranking alone. Using this systematic prioritization framework, *EPHA5* was selected for independent validation in tissue, saliva and serum samples obtained from patients with OSCC, OPML and healthy controls to evaluate its potential as a candidate non-invasive epigenetic biomarker for OSCC.

## 2 Materials and methods

### 2.1 Sample collection

The study was conducted in two sequential phases consisting of a genome-wide discovery phase followed by an independent validation phase. During the discovery phase, paired tumor and adjacent normal tissue specimens were collected from six patients with primary OSCC undergoing primary surgical treatment at the Department of Otorhinolaryngology-Head and Neck Surgery, Istanbul University Faculty of Medicine. These samples were used for genome-wide DNA methylation and whole-transcriptome gene expression profiling.

For the independent validation phase, paired tumor and adjacent normal tissue specimens together with matched saliva and serum samples were collected from an independent cohort of 21 patients with primary OSCC treated at the same institution. OPML samples were obtained from 22 patients treated at the Department of Oral and Maxillofacial Surgery, Istanbul University Faculty of Dentistry. Healthy control samples were obtained from individuals without malignant or premalignant oral lesions who underwent uvulopalatopharyngoplasty at the Department of Otorhinolaryngology-Head and Neck Surgery, Istanbul University Faculty of Medicine. Oral mucosal tissue specimens from four healthy individuals were included in the genome-wide discovery analyses, whereas oral mucosa, saliva and serum samples from an additional 16 healthy individuals comprised the healthy control group for the independent validation cohort. Only patients with primary, previously untreated OSCC were eligible for inclusion. Patients with recurrent disease, previous chemotherapy or radiotherapy, or a history of a second primary malignancy were excluded. Healthy controls had no history of malignant or premalignant oral lesions or active inflammatory oral diseases at the time of sample collection. An *a priori* power analysis was performed during the study design using G*Power version 3.1 (Heinrich Heine University Düsseldorf, Germany). Using a significance level (α) of 0.05 and a statistical power (1-β) of 80%, the minimum required sample size was calculated as 16 subjects per group. The final validation cohort met this predefined minimum sample size requirement in all study groups.

The study protocol was approved by the Ethics Committee of Istanbul Faculty of Medicine (Approval No. 2013/1470), and written informed consent was obtained from all participants before sample collection.

All blood and saliva samples were collected before surgical intervention. Unstimulated whole saliva samples were collected after participants had refrained from eating and drinking for at least one hour. Before collection, participants rinsed their mouths with drinking water for one minute and were then instructed to accumulate saliva within the oral cavity for approximately 5 minutes until approximately 5 mL of saliva was obtained. Saliva samples were diluted 1:1 with isotonic saline, centrifuged at 1,950 rpm for 5 minutes, and the supernatant was discarded. Peripheral blood samples were centrifuged at 1,950 rpm for 30 minutes, and serum was aliquoted into 1 mL fractions. All tissue, saliva and serum specimens were stored at -80 °C until DNA and RNA extraction.

### 2.2 Cytopathological evaluation

Because of the limited availability of tissue specimens, adjacent normal oral mucosa obtained from patients with premalignant or malignant oral lesions, together with normal oral mucosa from healthy individuals, was evaluated using the imprint cytology technique. Briefly, the freshly cut tissue surface was gently pressed onto clean glass slides several times to obtain cellular imprints. The slides were fixed in 96% ethanol for at least 15 minutes, stained using the Papanicolaou (Pap) method and examined under a light microscope. The cytological evaluation included assessment of nuclear and cellular morphology, nuclear-to-cytoplasmic ratio, nuclear pleomorphism, hyperchromasia, mitotic activity, abnormal mitotic figures and nucleolar prominence. The evaluated morphological features including loss of epithelial polarity, disturbed epithelial stratification, maturation abnormalities within the basal and spinous layers, dyskeratosis, basal cell hyperplasia, broad epithelial rete ridges, papillary branching, increased cellular density, and carcinoma *in situ* extending from the basal layer toward the epithelial surface were also evaluated. Pathological alterations were identified in tumor tissues, whereas adjacent normal tissues or healthy control mucosa showed no pathological abnormalities.

### 2.3 Nucleic acid isolation and processing

Genomic DNA and total RNA were isolated from the collected specimens according to the protocols described below. Nucleic acid concentration and purity were determined using a NanoDrop 2000c spectrophotometer (Thermo Fisher Scientific, MA, USA). All DNA and RNA samples were stored at -80 °C until molecular analyses.

#### 2.3.1 DNA isolation and bisulfite modification

For the genome-wide DNA methylation analyses, frozen tumor specimens were microdissected and hematoxylin and eosin (H&E)-stained sections were reviewed to confirm a tumor cell content of at least 70%. Genomic DNA was isolated from paired tumor and adjacent normal tissue specimens obtained from six patients with OSCC and normal oral mucosa specimens from four healthy individuals included in the discovery cohort, as well as from tissue, saliva and serum samples obtained from 21 patients with OSCC, 22 patients with OPML and 16 healthy controls in the validation cohort, using phenol/chloroform extraction following proteinase K digestion at 37 °C for 4–18 hours. Bisulfite conversion and purification of discovery cohort samples were performed using the EpiTect Bisulfite Kit (Qiagen, Hilden, Germany), whereas validation cohort samples were processed using the EZ DNA Methylation-Gold Kit (Zymo Research, CA, USA) according to the manufacturers’ instructions.

#### 2.3.2 RNA isolation and cRNA/cDNA synthesis

Total RNA was isolated from paired tumor and adjacent normal tissue specimens obtained from six patients with OSCC and normal oral mucosa specimens from four healthy individuals included in the discovery cohort using the Norgen Total RNA Purification Kit (Norgen Biotek Corp., Ontario, Canada) according to the manufacturer’s instructions. For genome-wide gene expression analyses, cRNA synthesis and labeling were performed using the Epicentre TargetAmp Nano Labeling Kit (Epicentre Biotechnologies, USA). For the validation cohort, total RNA was extracted from tissue, saliva and serum samples obtained from 21 patients with OSCC, 22 patients with OPML and 16 healthy controls using the same protocol. Complementary DNA (cDNA) was synthesized using the SensiFAST cDNA Synthesis Kit (Bioline, UK).

### 2.4 DNA methylation analysis

#### 2.4.1 Genome-wide DNA methylation profiling

Genome-wide DNA methylation profiling was performed using the Illumina HumanMethylation450 BeadChip (Illumina, CA, USA) on bisulfite-converted DNA obtained from paired tumor and adjacent normal tissue specimens from six patients with OSCC and normal oral mucosa specimens from four healthy individuals included in the discovery cohort. Briefly, 200 ng of bisulfite-converted DNA was processed according to the manufacturer’s standard Infinium HD methylation assay protocol, including whole-genome amplification, enzymatic fragmentation, hybridization to the BeadChip, single-base extension, fluorescent staining, and scanning. Arrays were scanned using the Illumina iScan System and raw intensity data were extracted and subjected to quality control using Illumina GenomeStudio software.

#### 2.4.2 Validation of *EPHA5* promoter methylation by quantitative methylation-specific PCR

QMSP was performed to evaluate *EPHA5* promoter methylation in paired tumor and adjacent normal tissue specimens, saliva and serum samples obtained from 21 patients with OSCC, paired lesion and adjacent normal tissue specimens, saliva and serum samples obtained from 22 patients with OPML, and oral mucosal tissue, saliva and serum samples obtained from 16 healthy controls. The promoter region and CpG island of the *EPHA5* gene were identified using the University of California, Santa Cruz (UCSC) Genome Browser (http://genome.ucsc.edu/). Methylation-specific primers and probe targeting the promoter region of *EPHA5* were designed using MethPrimer (10) and Primer-BLAST (11). *ACTB* primers and probe served as the internal reference (12). The primer and probe sequences were as follows: *EPHA5*: sense, 5’-TTAGTAGCGGTAAGCGGTA-3’; antisense, 5’-ACGCGAATCCTCCGTTTAA-3’; probe, 5’-FAM-CGATGTGACGATAAGGTTGTTGAT–TAMRA-3’; and *ACTB*: sense, 5’-TGGTGATGGAGGAGGTTTAGTAAGT-3’; antisense, 5’-AACCAATAAAACCTACTCCTCCCTTAA-3’; probe, 5’-FAM-ACCACCACCCAACACACAATAACAAACACA-TAMRA-3’. Each QMSP reaction was performed in a final volume of 20 μL containing 600 nM of each primer, 200 nM fluorescent probe, 0.6 U Platinum Taq DNA polymerase, 200 μM of each dNTP, 1xPCR buffer, 1xROX reference dye, and 3 μL of bisulfite-converted DNA. PCR amplification was performed using the LightCycler480 System (Roche Diagnostics GmbH, Mannheim, Germany) with an initial denaturation step at 95 °C for 5 minutes, followed by 50 cycles at 95 °C for 15 seconds, 64 °C for 1 minute, and 72 °C for 15 seconds. Fluorescence data were analyzed using the Absolute Quantification/Fit Points module of the LightCycler 480 software (Roche Diagnostics GmbH, Mannheim, Germany). Each sample was analyzed at least in duplicate. Genomic DNA isolated from lymphocytes of a healthy individual was methylated *in vitro* using excess SssI methyltransferase and S-adenosylmethionine (Zymo Research, CA, USA) to generate fully methylated control DNA. Following bisulfite modification, serial dilutions (1:10-1:10,000) of the fully methylated DNA were used to construct standard curves for each experiment. A common fluorescence threshold was applied to both *EPHA5* and *ACTB*, and relative DNA methylation levels were calculated as the ratio of *EPHA5* to *ACTB* amplification signals and expressed as (*EPHA5*/*ACTB*) x 100.

### 2.5 Gene expression analysis

#### 2.5.1 Genome-wide gene expression profiling

Genome-wide gene expression profiling was performed using the Illumina HumanHT-12 v4 Expression BeadChip (Illumina, CA, USA) on paired tumor and adjacent normal tissues from six OSCC patients together with normal oral mucosa from four healthy individuals. Briefly, 500 ng of labeled cRNA was hybridized to the BeadChips according to the manufacturer’s instructions. Following hybridization, washing, fluorescent labeling and scanning, raw expression data were extracted for downstream analyses. Based on the integrated genome-wide DNA methylation and transcriptomic analyses, *EPHA5* was prioritized for independent validation from a panel of 51 candidate genes by integrating concordant DNA methylation and gene expression alterations with pathway enrichment, biological relevance and translational applicability (**Supplementary Table 1**).

#### 2.5.2 Validation of *EPHA5* gene expression by quantitative real-time PCR

Primers for *EPHA5* were designed using Primer-BLAST (https://www.ncbi.nlm.nih.gov/tools/primer-blast/). The primer sequences were as follows: (sense, 5’-TGTGACCGATGAACCTCCCA-3’; antisense, 5’-GAACCCAGGTCTGCACACTT-3’). The *ACTB* gene was used as the endogenous reference control and the primer sequences for *ACTB* were adopted from a previous study (12). Quantitative Real-Time PCR (QRT-PCR) analyses were performed using the LightCycler480 System (Roche Diagnostics GmbH, Mannheim, Germany) on tissue and body fluid samples obtained from 21 OSCC patients, 22 OPML patients and 16 healthy individuals. PCR amplification consisted of an initial denaturation at 95 °C for 5 minutes, followed by 45 cycles of 95 °C for 15 seconds, 60 °C for 1 minute, 95 °C for 10 seconds, and 55 °C for 20 seconds. Relative gene expression levels were calculated using the 2^-ΔΔCt^ method (13), and genes with an absolute fold-change of ≥2 were considered differentially expressed.

To minimize potential analytical bias, all molecular analyses were performed using laboratory-coded samples. Investigators performing the QMSP and QRT-PCR assays had access only to laboratory identification codes and were blinded to the pathological diagnosis and clinicopathological characteristics of the study participants during the experimental procedures. Statistical analyses were performed independently by a different investigator.

### 2.6 Data processing and analysis

The genome-wide DNA methylation and gene expression datasets generated in the discovery cohort have been deposited in the Gene Expression Omnibus (GEO) under accession numbers GSE208065 (DNA methylation), GSE208067 (gene expression), and GSE208068 (SuperSeries).

#### 2.6.1 DNA methylation data analysis

Raw intensity data generated from the Illumina HumanMethylation450 BeadChip were processed in the R environment using the ChAMP package (14, 15). First, the raw intensity values from *.idat files were imported into R as β-values (16, 17). Quality-control filtering excluded probes with detection p-values >0.01, bead counts <3, non-CpG probes, probes located on sex chromosomes, probes overlapping polymorphic regions (18), and probes mapping to multiple genomic locations (19). Beta-Mixture Quantile (BMIQ) normalization was subsequently applied (20). Potential sources of technical variation were assessed using singular value decomposition (SVD) analysis (21), and batch effects were corrected using the ComBat algorithm when required (22, 23). Genome-wide DMP analysis was performed descriptively to characterize the overall DNA methylation landscape across the array and was not used for candidate prioritization. Differentially methylated regions (DMRs) were identified using the Probe-Lasso algorithm (24), implemented in the ChAMP package. Candidate regions were required to contain at least three CpG probes. Candidate DMRs were identified when they showed a nominal regional p-value < 0.1 and an absolute mean difference in methylation beta values (|Δβ| ≥0.10) between the two study groups. To increase the robustness of the region-level findings, only regions supported by multiple neighboring CpG probes and demonstrating a consistent direction of methylation change across samples were retained. Adjusted p-values were not used as a selection criterion for DMR identification because of the exploratory nature of the discovery analysis and the limited sample size relative to the large number of CpG sites tested. DMRs were annotated according to the hg19 human reference genome.

#### 2.6.2 Gene expression data analysis

Genome-wide gene expression data were analyzed in the R environment (v3.5.1) using the limma and lumi packages (15, 25, 26). Raw intensity data were imported from Illumina *.idat files, followed by background correction, quantile normalization and probe annotation using the corresponding Illumina Bead Manifest file (support.illumina.com/sequencing/sequencing_software/basespace.html). Differential expression analysis was performed using linear models implemented in the limma package with empirical Bayes moderation. Multiple-testing correction was performed across the entire gene expression array using the Benjamini-Hochberg (BH) procedure, and genes with an adjusted FDR <0.05 were considered differentially expressed. Because the BH procedure enforces monotonicity of adjusted p-values across ranked hypotheses, identical adjusted p-values may occur for multiple genes. Hierarchical clustering of differentially expressed genes was performed using Pearson correlation distance (1-cor(x,y)) and complete linkage clustering. Heatmaps were generated using the ComplexHeatmap package in R (27). An analogous clustering approach was applied to differentially methylated probes using normalized ß-values.

#### 2.6.3 Pathway enrichment analysis for candidate prioritization

To identify dysregulated biological pathways associated with OSCC, pathway enrichment analyses were performed separately using differentially methylated and differentially expressed genes. Active subnetwork-based pathway enrichment analysis was conducted using the PathfindR package, which integrates protein-protein interaction (PPI) networks with pathway annotations to identify biologically connected gene subnetworks (28). Genes associated with DMRs (**Supplementary Table 2**) and DEGs (**Supplementary Table 3**) were subsequently analyzed using the Kyoto Encyclopedia of Genes and Genomes (KEGG) database (https://www.kegg.jp/kegg/) to identify significantly enriched signaling pathways (29). Protein-protein interaction networks were constructed and visualized using the STRING database (https://string-db.org/) to investigate functional interactions among the prioritized candidate genes. Pathways consistently identified in both methylation- and transcriptome-based enrichment analyses were considered high-confidence candidates for subsequent prioritization. The final candidate selection considered consistency across multiple analytical approaches together with biological relevance and feasibility for independent experimental validation. Candidate genes were therefore prioritized by integrating promoter methylation status, gene expression changes, pathway enrichment results, protein-protein interaction analyses, and translational applicability rather than statistical ranking alone.

### 2.7 Statistical analysis

All statistical analyses for the validation cohort were performed using the IBM SPSS Statistics for Windows, version 31.0 (IBM Corp., Armonk, NY, USA) (30). Data distribution was assessed using the Shapiro-Wilk test. Comparisons between groups were performed using the independent-samples t test for normally distributed variables and the Mann-Whitney U test or Wilcoxon signed-rank test for non-normally distributed variables, as appropriate. Correlations between continuous variables were evaluated using Spearman’s rank correlation coefficient. Associations between clinicopathological variables and *EPHA5* methylation or gene expression status were investigated using univariate and multivariate logistic regression analyses. Odds ratios (ORs) and 95% confidence intervals (Cls) were calculated. To minimize model overfitting, only biologically relevant variables were included in the multivariate logistic regression analyses. Receiver operating characteristic (ROC) curve analysis was performed to evaluate the diagnostic performance of *EPHA5* methylation and gene expression. The area under the curve (AUC), optimal cut-off values, sensitivity, specificity, and corresponding 95% confidence intervals (95% Cls) were calculated. Statistical significance was defined as p <0.05.

The overall study workflow is illustrated in **Figure 1**.

**Figure 1:**
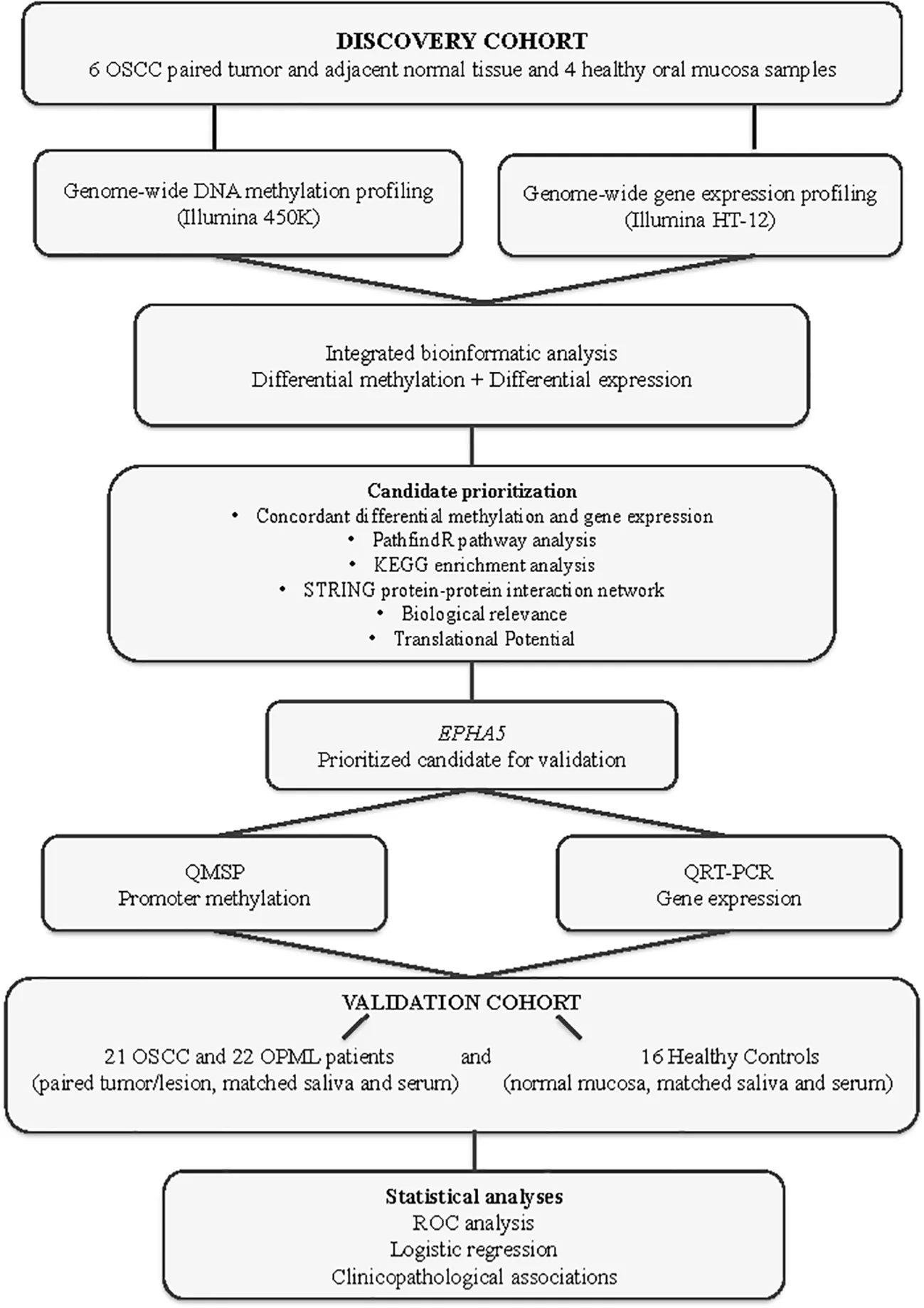
Schematic overview of the study workflow. Genome-wide DNA methylation and transcriptomic analyses performed in the discovery cohort were integrated for candidate prioritization using pathway enrichment, protein-protein interaction analysis, biological relevance and translational potential. *EPHA5* was subsequently selected for independent validation by QMSP and QRT-PCR in the validation cohort comprising paired tissue, saliva and serum samples.

## 3 Results

### 3.1 Demographic, clinical and pathological characteristics

The study cohort comprised 27 patients with OSCC, including a genome-wide discovery cohort of six patients and an independent validation cohort of 21 patients, together with 22 patients with OPML and 16 healthy controls. Genome-wide DNA methylation and transcriptomic profiling were performed in the discovery cohort, followed by independent validation of *EPHA5* promoter methylation and gene expression by QMSP and QRT-PCR in paired tissue, saliva, and serum samples obtained from the validation cohort (**Figure 1**).

The independent validation cohort comprised 21 patients with OSCC (mean age, 61.95 ± 11.59 years; range, 38–83 years), of whom 66.7% (14/21) were male. Primary tumor sites included the buccal mucosa (38.1%, 8/21), tongue (28.6%, 6/21), floor of the mouth (23.8%, 5/21), retromolar trigone (4.8%, 1/21), and hard palate (4.8%, 1/21). According to the TNM classification, 9.5% (2/21) of patients had stage I disease, 28.6% (6/21) stage II, 19% (4/21) stage III, and 42.9% (9/21) stage IV disease. Current smoking and alcohol consumption were reported in 57.1% (12/21) and 9.5% (2/21) of patients, respectively. Lymph node metastasis was present in 42.9% (9/21) of cases and disease recurrence occurred in 38.1% (8/21) during follow-up. The clinicopathological characteristics of the validation cohort and the distribution of *EPHA5* methylation and expression status are summarized in **Table 1**.

**Table 1:** Clinicopathological characteristics of the OSCC validation cohort and distribution of EPHA5 methylation and gene expression status.

Expression/ Methylation Results Clinical Parameters		Total	Tumor Methylation n (%)	p	Saliva Methylation n (%)	p	Decreased Expression n (%)	p	Increased Expression n (%)	p	Meth-based Expression Loss n (%)	p
**OSCC Patients n (%)**		**21 (100)**	**14 (66.7)**		**10 (47.6)**		**9 (42.9)**		**5 (23.8)**		**6 (28.6)**	
**Age (mean + SD)**	61.95±11.59		64.71±11.59		63.4±11.59		66.4±11.59		53.8±14.35		65.5±11.58	
**Gender**	Female	7 (33.3)	4 (28.6)	0.174	5 (50)	0.036*	3 (33.3)	0.381	1 (20)	0.400	2 (33.3)	0.533
	Male	14 (66.7)	10 (71.4)		5 (50)		6 (66.7)		4 (80)		4 (66.7)	
**Anatomical Region**	Floor of Mouth	5 (23.8)	1 (7.1)	0.097	3 (30)	0.536	3 (33.3)	0.614	1 (20)	0.467	1 (16.6)	0.200
	Buccal	8 (38.1)	8 (57.3)		5 (50)		4 (44.5)		2 (40)		4 (66.7)	
	Retromolar Trigone	1 (4.7)	1 (7.1)		0		0		0		0	
	Tongue	6 (28.7)	3 (21.4)		1 (10)		2 (22.2)		2 (40)		1 (16.6)	
	Hard Palate	1 (4.7)	1 (7.1)		1 (10)		0		0		0	
**Overall Stage**	I	2 (9.5)	1 (7.1)	0.957	0	0.064	1 (11.2)	0.621	1 (20)	0.900	0	0.800
	II	6 (28.7)	4 (28.6)		1 (10)		2 (22.2)		1 (20)		1 (16.6)	
	III	4 (19)	4 (28.6)		2 (20)		3 (33.3)		1 (20)		3 (50.1)	
	IV	9 (42.8)	5 (35.7)		7 (70)		3 (33.3)		2 (40)		2 (33.3)	
**Clinical Stage**	Early Stage	8 (38.1)	5 (35.7)	0.814	1 (10)	0.058	3 (33.3)	0.548	2 (40)	0.400	1 (16.6)	0.667
	Advanced Stage	13 (61.9)	9 (64.3)		9 (90)		6 (66.7)		3 (60)		5 (83.4)	
**Differentiation**	Well	5 (23.8)	4 (28.6)	0.452	3 (30)	0.220	1 (11.2)	0.222	0	–	1 (16.6)	1.000
	Moderately	16 (76.2)	10 (71.4)		7 (70)		8 (88.8)		5 (100)		5 (83.4)	
	Poorly	0	0		0		0		0		0	
**Lymph Node Metastasis**	(-)	12 (57.1)	9 (64.3)	0.312	5 (50)	0.380	4 (44.5)	1.000	2 (40)	0.400	3 (50)	0.400
	(+)	9 (42.9)	5 (35.7)		5 (50)		5 (55.5)		3 (60)		3 (50)	
**Smoking**	(-)	9 (42.9)	6 (42.7)	0.875	4 (40)	0.617	6 (66.7)	0.167	1 (20)	0.800	4 (66.7)	0.533
	(+)	12 (57.1)	8 (57.3)		6 (60)		3 (33.3)		4 (80)		2 (33.3)	
**Alcohol**	(-)	19 (90.5)	13 (92.9)	0.979	8 (80)	0.063	9 (100)	–	5 (100.0)	–	6 (100)	–
	(+)	2 (9.5)	1 (7.1)		2 (20)		0		0		0	
**Recurrence**	(-)	13 (61.9)	7 (50)	0.419	5 (50)	0.142	6 (66.7)	0.548	3 (60)	0.800	3 (50)	0.400
	(+)	8 (38.1)	7 (50)		5 (50)		3 (33.3)		2 (40)		3 (50)	
**Treatment**	Surgery	6 (28.7)	4 (28.6)	0.845	1 (10)	0.097	3 (33.3)	1.000	1 (20)	1.000	2 (33.3)	0.133
	Surgery + RT	14 (66.6)	9 (64.3)		8 (80)		6 (66.7)		4 (80)		4 (66.7)	
	Surgery + CRT	1 (4.7)	1 (7.1)		1 (10)		0		0		0	

meth, methylation; exp, expression; meth-based exp, methylation-based expression; bold values and * indicate statistical significance (p < 0.05).

The OPML cohort comprised 22 patients (mean age 50.2 ± 14.0 years; median age 48 years; range 31–83 years), including erosive oral lichen planus (EOLP, n=8), oral lichen planus (OLP, n=8) and oral lichenoid dysplasia (OLD, n=6). Overall, 59.1% (13/22) of patients were female, 45.5% (10/22) were smokers, 22.7% (5/22) reported alcohol consumption and 13.6% (3/22) had a family history.

Among the OPML subgroups, patients with EOLP had a mean age of 44.5 years and were predominantly female (87.5%), whereas patients with OLP had a mean age of 50 years, with females accounting for 62.5% of the subgroup. In contrast, patients with OLD were older (mean age, 58 years), predominantly male (83.3%) and exhibited the highest frequencies of smoking (83.3%) and alcohol consumption (66.7%).

The healthy control group comprised 16 individuals (mean age, 54.4 ± 8.2 years; range, 44–75 years), of whom 75.0% (12/16) were male and 25.0% (4/16) were female. Current smoking was reported by seven individuals (43.8%), alcohol consumption by three (18.8%), and a family history of cancer by two (12.5%).

### 3.2 Genome-wide methylation and transcriptomic profiling

#### 3.2.1 Genome-wide gene expression profile of the OSCC group

Genome-wide transcriptomic analysis identified 661 upregulated and 577 downregulated genes (BH-adjusted FDR <0.05) in OSCC tumors relative to matched adjacent normal tissues (**Figure 2A**).

**Figure 2:**
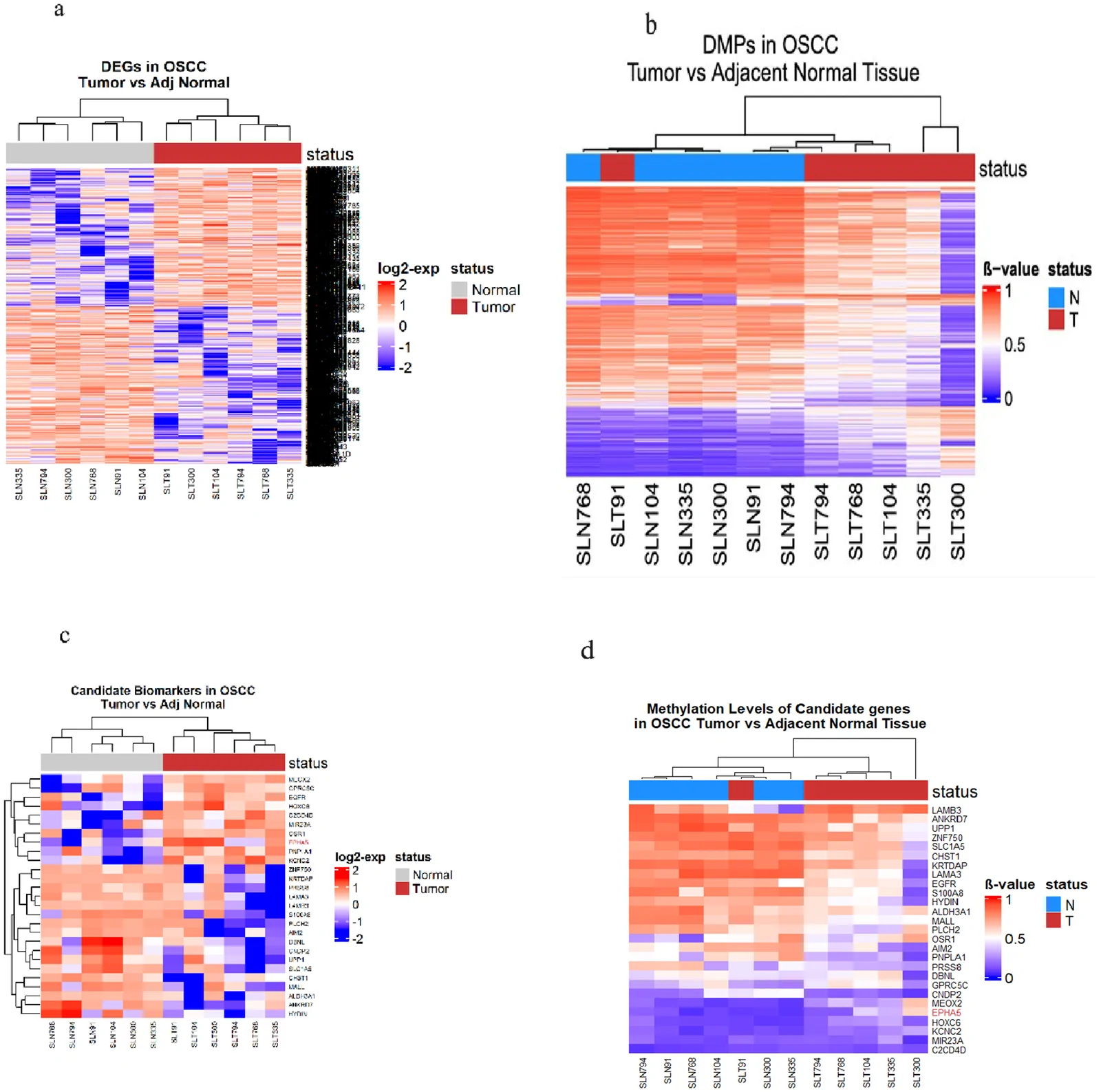
Genome-wide methylation and expression profiling in the OSCC discovery cohort. **(A)** Heatmap of differentially expressed genes (DEGs) identified between OSCC tumors and matched adjacent normal tissues (n=1,238). **(B)** Differentially methylated regions (DMRs) identified between OSCC tumors and matched adjacent normal tissues. **(C)** Heatmap of candidate epigenetically deregulated genes identified through integrated DNA methylation and transcriptomic analysis based on gene expression profiles. **(D)** Heatmap of candidate epigenetically deregulated genes identified through integrated DNA methylation and transcriptomic analysis based on DNA methylation profiles. DMR, differentially methylated region; N, adjacent normal tissue; T, tumor tissue; SLN, adjacent normal sample code; SLT, tumor sample code.

#### 3.2.2 Genome-wide DNA methylation profile of the OSCC group

Genome-wide DNA methylation profiling revealed a predominance of global hypomethylation in OSCC, with 34,223 hypomethylated and 12,835 hypermethylated differentially methylated probes (DMPs). Consistent with previous reports describing the DNA methylation landscape of cancer, 4,236 hypermethylated DMPs (33%) were located within promoter regions (31). Exploratory region-based analysis identified 2,431 differentially methylated regions (DMRs) corresponding to 1,776 genes (**Figure 2B**). Among these, 693 genes were associated with hypermethylated DMRs, whereas 1,738 genes were associated with hypomethylated DMRs, consistent with the predominance of global hypomethylation while highlighting a subset of promoter-specific hypermethylation events with potential biological relevance. To prioritize biologically relevant candidate epigenetic biomarkers for independent validation, genome-wide DNA methylation and transcriptomic datasets were subsequently integrated, yielding a panel of candidate genes for downstream prioritization.

### 3.3 Candidate biomarker identification

This integrative analysis generated a panel of 51 candidate genes prioritized through the integration of genome-wide DNA methylation and transcriptomic data. Differentially expressed genes were identified using a BH-adjusted FDR <0.05, whereas methylation candidates were derived from the exploratory region-level DMR analysis according to predefined biological and technical selection criteria (**Supplementary Table 1**). Active-subnetwork pathway enrichment analysis using PathfindR was performed separately for differentially expressed genes and genes associated with differentially methylated regions to identify biologically disrupted pathways in OSCC (**Supplementary Table 4**). Among these candidates, 10 genes displayed promoter hypermethylation accompanied by reduced gene expression, whereas 17 genes showed promoter hypomethylation together with increased expression, consistent with epigenetic regulation of transcription. The remaining candidates were retained based on biologically relevant methylation or gene expression alterations together with pathway enrichment, protein-protein interaction analyses, biological plausibility, and translational relevance.

Among the 51 candidate genes, *EPHA5* was prioritized as a biologically and translationally relevant candidate for independent validation based on the integrated multi-omics evidence rather than the magnitude of differential methylation or gene expression alone. In the discovery cohort, *EPHA5* exhibited significant promoter hypermethylation (Δß = 0.1006, p = 0.002) together with reduced gene expression (logFC = -0.089, BH-adjusted FDR <0.05), consistent with an epigenetic contribution to transcriptional regulation (**Figures 2C, D**; **Supplementary Table 1**). Moreover, *EPHA5* has established biological roles in cancer-related signaling pathways and has previously been reported to undergo methylation-mediated transcriptional silencing in several malignancies (32–36). Despite this evidence, its epigenetic and diagnostic significance in OSCC remains largely unexplored. Accordingly, *EPHA5* was prioritized by integrating promoter methylation status, gene expression changes, pathway enrichment analyses, protein-protein interaction analyses, biological plausibility, and translational relevance rather than statistical ranking or effect size alone.

#### 3.3.1 *EPHA5* as a candidate non-invasive epigenetic biomarker

To validate the candidate prioritized through the genome-wide discovery analysis, *EPHA5* promoter methylation and gene expression were independently evaluated in tissue and matched body fluid samples obtained from patients with OSCC (n = 21), OPML (n = 22) and healthy controls (n = 16) using QMSP and QRT-PCR, respectively.

*EPHA5* promoter methylation was detected in 66.7% (14/21) of OSCC tumor tissues, 23.8% (5/21) of matched adjacent normal tissues, 14.3% (3/21) of serum samples and 47.6% (10/21) of saliva samples. In the OPML cohort, *EPHA5* methylation was detected in 54.5% (12/22) of lesion tissues, 36.4% (8/22) of matched adjacent normal tissues and 22.7% (5/22) of saliva samples, whereas no methylation was detected in serum samples (**Figure 3**). *EPHA5* methylation levels were significantly higher in both OSCC and OPML lesion tissues than in their matched adjacent normal tissues (OSCC, p = 0.003; OPML, p = 0.010; Wilcoxon signed-rank test).

**Figure 3:**
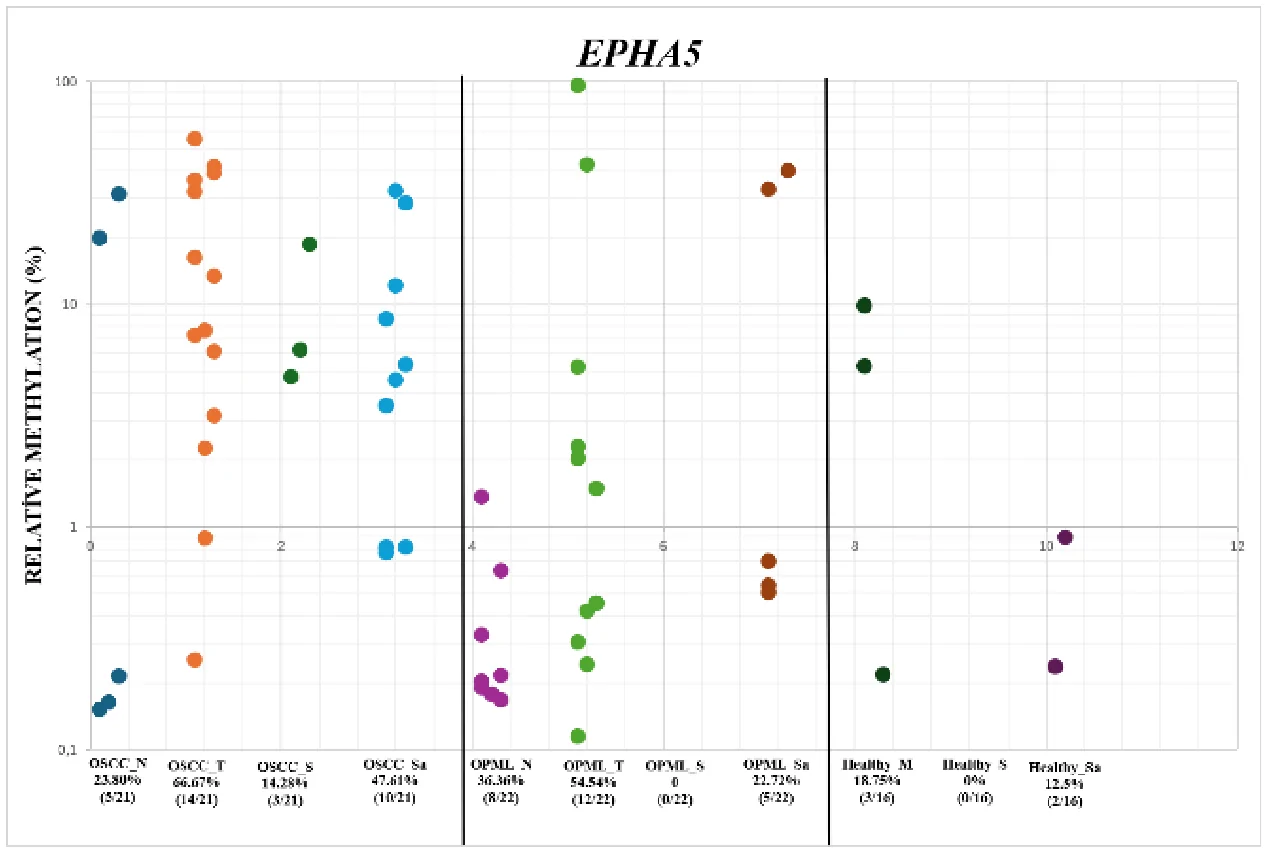
*EPHA5* promoter methylation profiles in OSCC, OPML and healthy control groups. Relative *EPHA5* methylation levels (%) in matched tumor, matched adjacent normal tissue, saliva, and serum samples. Samples with undetectable *EPHA5* methylation were omitted from the graph. N, adjacent normal tissue; M, mucosa; T, lesion/tumor tissue; S, serum; Sa, saliva.

Gene expression analysis demonstrated reduced *EPHA5* expression in 42.8% (9/21) of OSCC tumors relative to their matched adjacent normal tissues, whereas increased expression was observed in 23.8% (5/21) of tumors (**Figure 4A**). In contrast, *EPHA5* expression was increased in 36.4% (8/22) of OPML lesions, decreased in 27.3% (6/22), and remained unchanged in the remaining 36.4% (8/22) of cases (**Figure 4B**). Concordant promoter hypermethylation accompanied by reduced *EPHA5* expression was identified in six of 21 OSCC patients (28.6%), representing six of the 14 methylated tumors (42.9%), consistent with methylation-associated reduction in gene expression in a subset of tumors.

**Figure 4:**
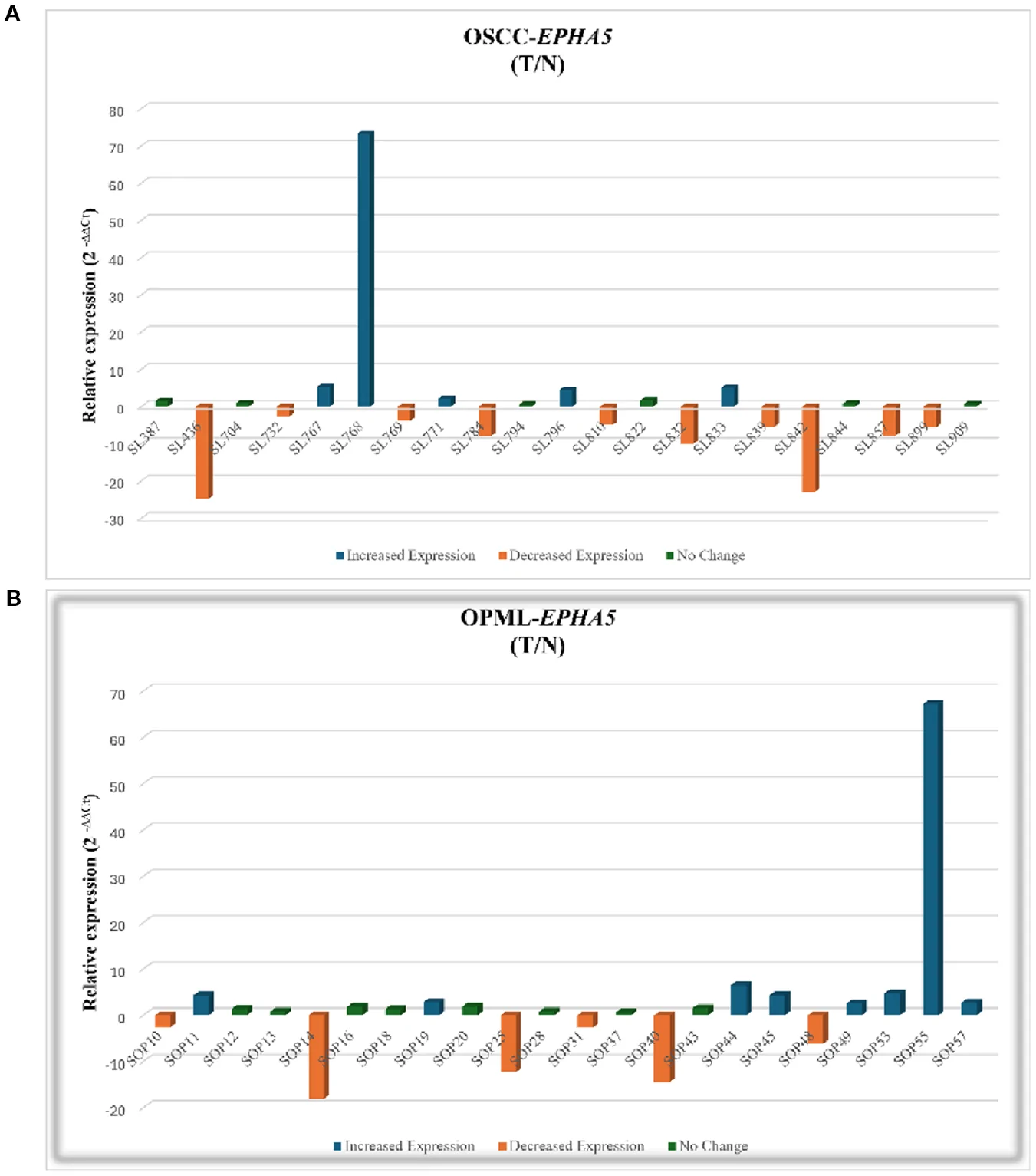
**(A)** Relative *EPHA5* gene expression (2^−ΔΔCt^) in paired OSCC tumor and matched adjacent normal tissue (T/N) from individual patients. Blue bars indicate increased expression, orange bars indicate decreased expression, and green bars indicate unchanged expression. **(B)** Relative *EPHA5* gene expression (2^−ΔΔCt^) in paired OPML lesion (T) and matched adjacent normal tissue (N)from individual patients. Expression levels are presented as lesion-to-normal (T/N) fold changes. Blue bars indicate increased expression, orange bars indicate decreased expression, and green bars indicate unchanged expression.

In the OPML cohort, methylation-associated loss of *EPHA5* expression was observed in 18.2% (4/22) of patients. Within the OPML subgroups, distinct methylation and expression patterns were observed. In patients with EOLP, *EPHA5* expression remained unchanged in 50% (4/8) of cases, whereas increased and decreased expression were each observed in 25% (2/8). Lesion and adjacent normal tissue methylation were both detected in 50% (4/8) of patients, salivary methylation was present in 37.5% (3/8) and methylation-associated loss of expression was identified in 12.5% (1/8). Among patients with OLP, *EPHA5* expression was increased in 50% (4/8) of cases, decreased in 12.5% (1/8) and remained unchanged in 37.5% (3/8) of cases. Lesion methylation was detected in 62.5% (5/8) of patients and adjacent normal tissue methylation in 37.5% (3/8), whereas salivary methylation was not detected. Methylation-associated loss of expression occurred in 12.5% (1/8) of patients. In the OLD subgroup, reduced *EPHA5* expression predominated, occurring in 50% (3/6) of patients, whereas increased and unchanged expression were observed in 33.3% (2/6) and 16.7% (1/6) of cases, respectively. Lesion methylation was detected in 50% (3/6) of patients, adjacent normal tissue methylation in 16.7% (1/6) and salivary methylation in 33.3% (2/6). Methylation-associated loss of *EPHA5* expression was identified in 33.3% (2/6) of patients.

#### 3.3.2 Clinical associations, diagnostic performance and survival analysis of *EPHA5*

Associations between *EPHA5* promoter methylation, gene expression, methylation-associated expression loss, and expression fold-change with clinicopathological characteristics were evaluated using non-parametric tests and logistic regression analyses. Overall, neither tumor *EPHA5* methylation status, gene expression status, methylation-associated expression loss, nor expression fold-change was significantly associated with the evaluated clinicopathological variables (all p > 0.05). These variables included age, sex, smoking status, alcohol consumption, tumor stage, lymph node involvement, recurrence, treatment modality and molecular subtype. Salivary methylation showed a significant association with sex (Mann-Whitney U test, p=0.036), whereas no significant correlation was observed between tumor and salivary methylation levels (Spearman’s rho =0.186, p=0.421). Although no significant associations were observed between smoking status, alcohol consumption, treatment modality and *EPHA5* methylation or expression parameters overall, salivary methylation showed a borderline association with alcohol consumption (Mann-Whitney U = 5.0, exact p=0.063).

Univariate logistic regression identified lymph node metastasis as the clinicopathological variable showing the strongest trend toward an association with reduced *EPHA5* expression (OR = 6.12, 95% CI: 0.83-45.1, p=0.075). A similar trend was observed in the multivariate model, although statistical significance was not reached (**Supplementary Tables 5**, **6**).

In the OPML cohort, no significant associations were observed between *EPHA5* expression, methylation-associated expression loss, expression fold-change or lesion methylation and the evaluated clinicopathological variables, including sex, smoking status, alcohol consumption, family history, histopathological subgroup or clinical outcome (all p > 0.05). The only statistically significant association was observed between lesion methylation and family history within the EOLP subgroup (Exact p = 0.048).

Receiver operating characteristic (ROC) analyses were performed to evaluate the diagnostic performance of *EPHA5* promoter methylation. Tumor methylation demonstrated limited discriminatory performance between OSCC and OPML, which did not reach statistical significance (AUC = 0.662, 95% CI: 0.493-0.832, p = 0.068), with 61.9% sensitivity and 72.7% specificity at the optimal cut-off value of 0.67 (**Figure 5A**). These findings indicate that *EPHA5* promoter methylation alone could not reliably discriminate OSCC from OPML. Salivary methylation showed limited discriminatory performance (AUC = 0.600, 95% CI: 0.423-0.776, p = 0.264), yielding 52.4% sensitivity and 77.3% specificity at a cut-off value of 0.06. In contrast, serum methylation (AUC = 0.571, 95% CI: 0.398-0.745, p=0.423) and adjacent normal tissue methylation (AUC = 0.468, 95% CI: 0.292-0.643, p=0.716) demonstrated poor discriminatory performance.

**Figure 5:**
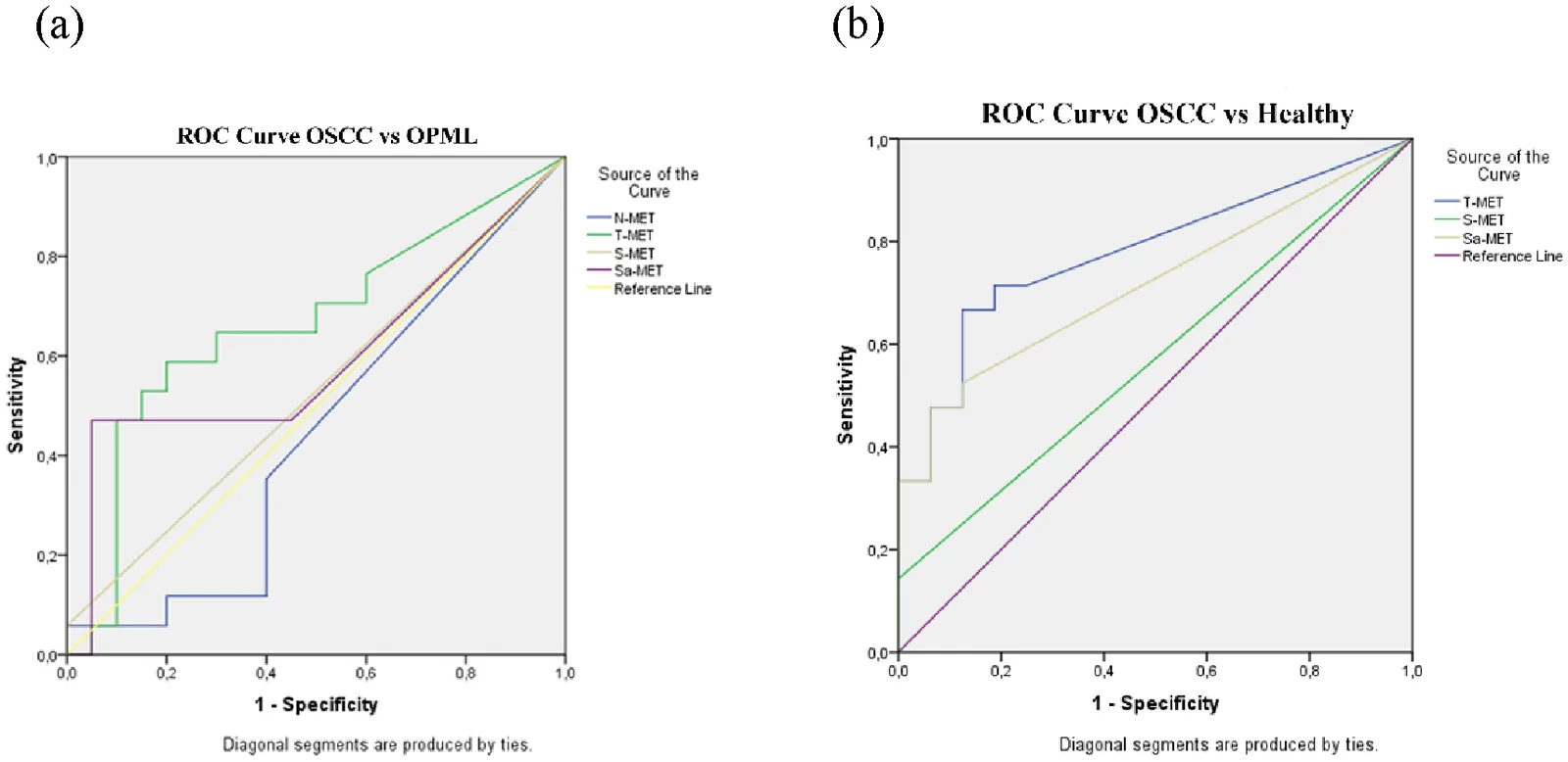
ROC curve analysis evaluating the diagnostic performance of *EPHA5* promoter methylation in different sample types. **(A)** ROC curves comparing OSCC patients (n=21) and OPML patients (n=22). **(B)** ROC curves comparing OSCC patients and healthy controls (n=16). ROC analyses were performed using *EPHA5* methylation levels in tumor/lesion tissue (T-MET), matched adjacent normal tissue (N-MET), saliva (Sa-MET) and serum (S-MET).

In comparisons between OSCC patients and healthy controls, tumor *EPHA5* methylation demonstrated moderate diagnostic performance (AUC = 0.780, 95% CI: 0.630-0.930, p = 0.004), with 71.4% sensitivity and 75.0% specificity at the optimal cut-off value of 0.0015 (**Figure 5B**). Salivary *EPHA5* methylation also significantly discriminated OSCC patients from healthy controls (AUC = 0.717, 95% CI: 0.553-0.882, p = 0.025), yielding 52.4% sensitivity and 87.5% specificity at a cut-off value of 0.0395. Serum methylation showed poor diagnostic performance (AUC = 0.571, 95% CI: 0.386-0.757, p=0.462).

Exploratory subgroup analyses were performed to evaluate the influence of anatomical tumor localization, tumor stage, smoking status, alcohol consumption and treatment modality on *EPHA5* methylation and expression parameters. Tumor methylation differed significantly according to anatomical localization (Fisher’s exact test, p = 0.007), with pairwise comparisons demonstrating higher methylation levels in buccal mucosal tumors than in tongue tumors (Mann-Whitney U = 8.0, exact p = 0.042). No significant differences were observed among anatomical sites with respect to *EPHA5* expression or methylation-associated expression loss (all exact p > 0.05). Likewise, no significant associations were identified between tumor stage and *EPHA5* methylation or gene expression parameters. However, salivary methylation demonstrated a trend toward higher levels in advanced-stage (Stages III-IV) compared with early-stage (Stages I-II) disease (Mann-Whitney U = 27.0, exact p = 0.058).

Exploratory ROC analyses were subsequently performed to evaluate the discriminatory performance of *EPHA5* methylation and gene expression according to tumor stage, recurrence and metastatic status (**Supplementary Figure 1**). Neither tumor methylation (AUC = 0.534, 95% CI: 0.270-0.798, p = 0.800) nor relative gene expression (AUC = 0.510, 95% CI: 0.253-0.766, p = 0.942) demonstrated significant discriminatory performance between early-stage and advanced-stage disease (**Supplementary Figures 1A**, **B**, respectively). Likewise, none of the evaluated tumor or salivary methylation or expression parameters demonstrated statistically significant discriminatory performance for metastatic status (**Supplementary Figures 1C, D, J**) and recurrence (**Supplementary Figures 1E, F, L**) (all p > 0.05), although salivary methylation showed a trend toward discriminating early-stage from advanced-stage disease (AUC = 0.740, 95% CI: 0.512-0.969, p = 0.070) with a sensitivity of 69.2% and a specificity of 87.5% (**Supplementary Figure 1G**) and moderate, but non-significant, discriminatory performance for recurrence (AUC = 0.688, 95% CI: 0.427-0.948, p = 0.158) with a sensitivity of 62.5% and a specificity of 84.6% (**Supplementary Figure 1K**), and metastatic status (AUC = 0.694, 95% CI: 0.440-0.948, p = 0.156) with a sensitivity of 71.4% and a specificity of 71.4% (**Supplementary Figure 1I**).

Kaplan–Meier survival analysis was performed in 19 OSCC patients with available follow-up data. The estimated median overall survival was 83.9 months (SE = 11.14; 95% CI: 62.04-105.72) (**Figure 6A**). Overall survival was further evaluated according to treatment modality, including surgery alone (n=5), surgery plus radiotherapy (n=13), and surgery plus chemoradiotherapy (n=1). No statistically significant differences in overall survival were observed among treatment groups according to the log-rank test (χ² = 2.891, p = 0.236), Breslow (χ² = 2.730, p = 0.255) or Tarone-Ware (χ² = 2.865, p = 0.239) tests (**Figure 6B**).

**Figure 6:**
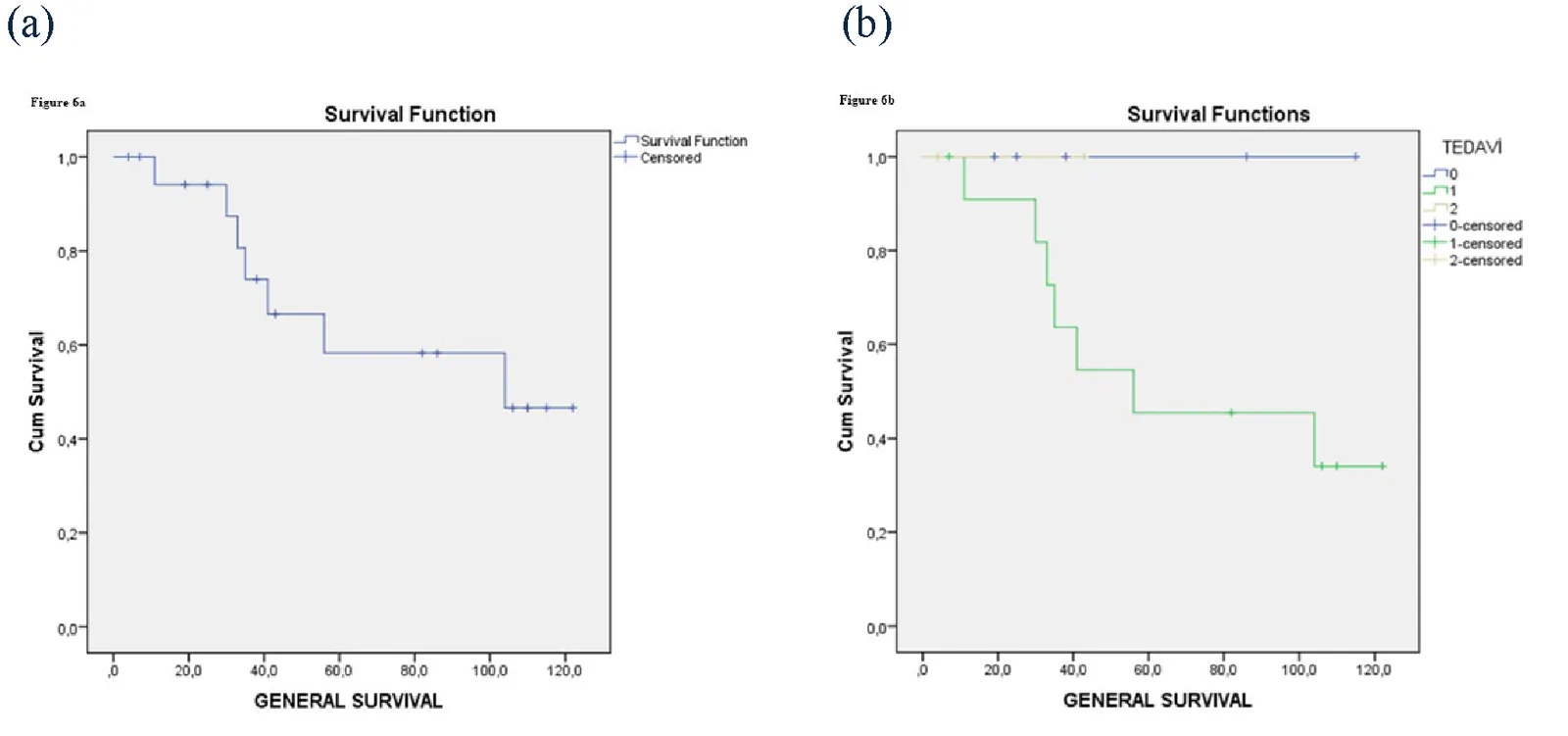
Kaplan-Meier overall survival analysis in patients with OSCC. **(A)** Overall survival curve for the OSCC cohort (n=19), **(B)** Overall survival according to treatment modality: 0=surgery alone; 1=surgery plus radiotherapy; 2= surgery plus chemoradiotherapy.

### 3.4 Pathway enrichment and protein-protein interaction analysis

Pathway enrichment analysis based on the KEGG database identified several significantly enriched biological pathways associated with the integrated genome-wide methylation and transcriptomic datasets (**Supplementary Table 4**). Among the significantly enriched pathways, the axon guidance pathway was highlighted because it included *EPHA5*, the candidate prioritized for independent validation and was among the most biologically relevant pathways identified through the integrated analysis (**Figure 7**).

**Figure 7:**
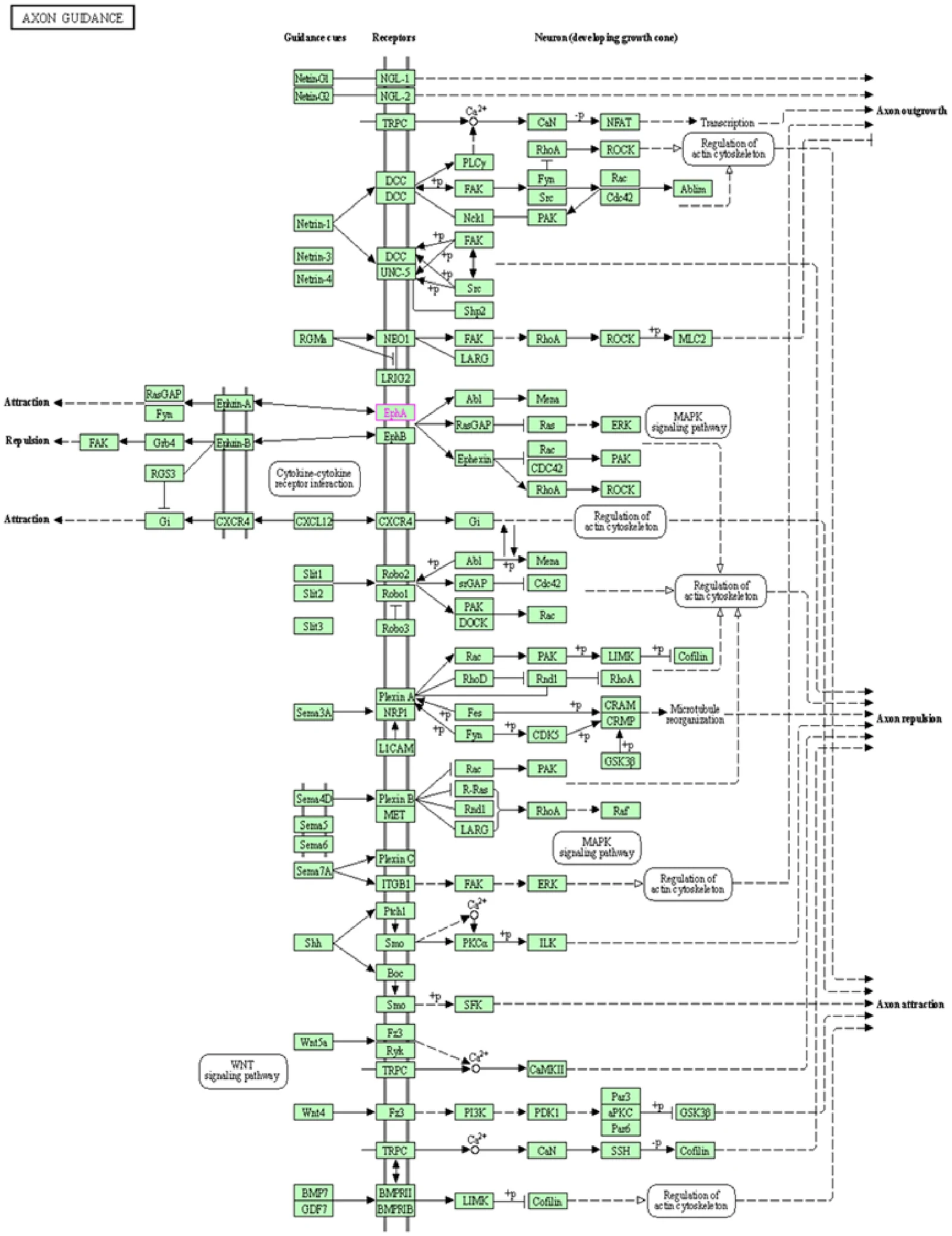
KEGG axon guidance pathway illustrating the position of *EPHA5* (highlighted in purple) within the associated signaling network. Adapted from the KEGG PATHWAY database, hsa04360 (Axon guidance), © Kanehisa Laboratories, with permission.

To further explore the functional context of EPHA5, protein-protein interaction analysis was performed using the STRING database. EPHA5 was predicted to interact with multiple proteins involved in Eph/ephrin signaling, cell adhesion, cytoskeletal organization and intracellular signaling, including EPHA1, EPHA4, EPHA6, EPHA7, EFNA1, EFNA2, EFNA3, EFNA5, EFNB2, FYN, SRC, RHOA, CBL, CDH1, PXN, STAT3, GJA1, ARHGDIA, ANLN and RTKN. The predicted interaction network demonstrated extensive connectivity between EPHA5, and proteins involved in Eph/ephrin signaling, cell signaling and cell adhesion (**Figure 8**).

**Figure 8:**
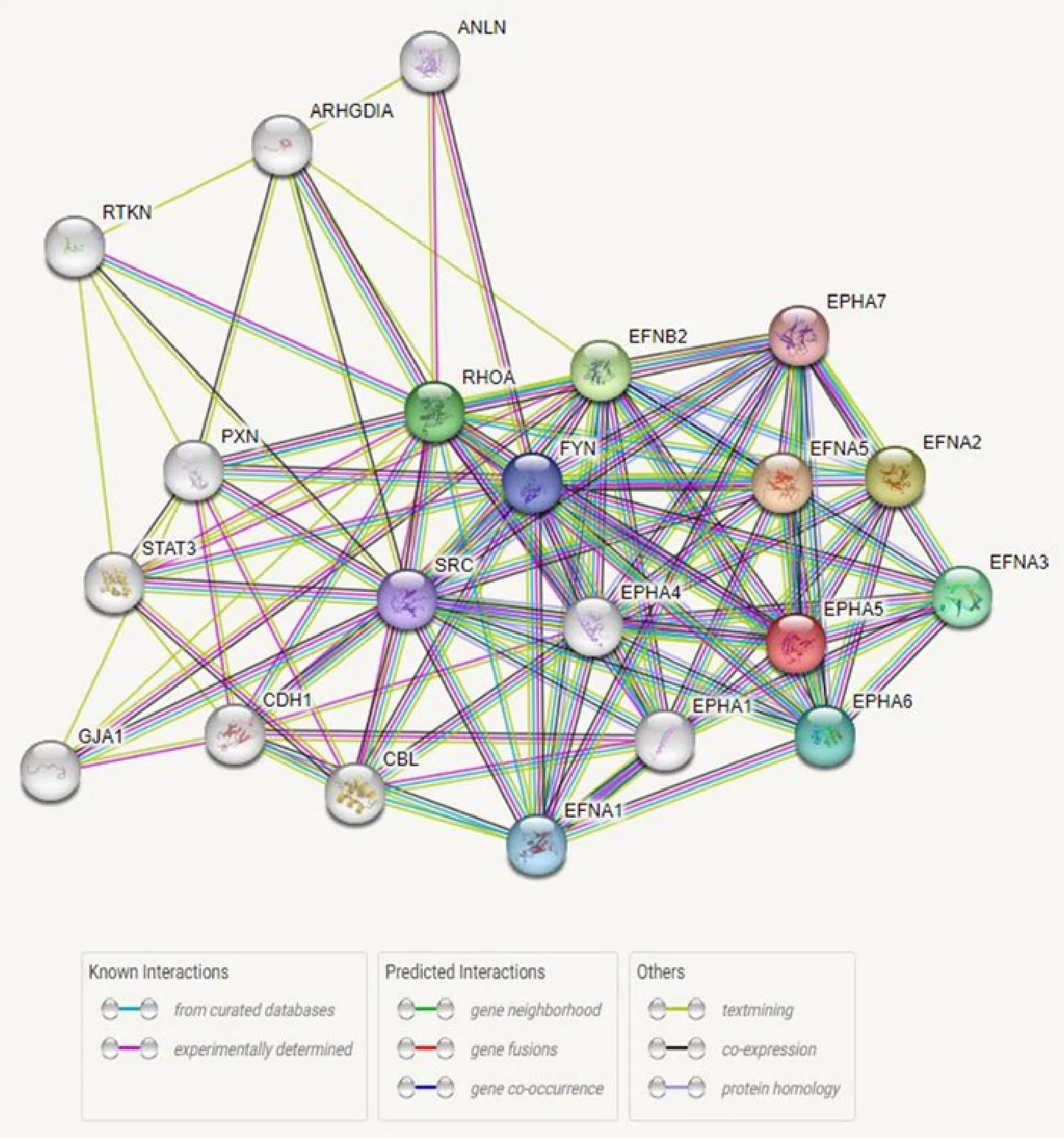
Protein-protein interaction (PPI) network of prioritized candidate genes generated using the STRING database. Nodes represent proteins, whereas edges indicate known and predicted functional associations derived from curated databases, experimental evidence, co-expression, text mining, gene neighborhood, gene fusion, gene co-occurrence and protein homology. The network illustrates the functional interactions of EPHA5 with proteins involved in Eph/ephrin signaling and related cellular pathways.

## 4 Discussion

Epigenetic alterations frequently precede detectable transcriptional, histopathological and clinical changes during carcinogenesis, making them attractive candidates for cancer detection, risk assessment and disease monitoring (9). Building upon this concept, the present study applied an integrated genome-wide DNA methylation and transcriptomic discovery strategy to identify epigenetically deregulated genes in OSCC. Rather than selecting candidate genes solely according to the magnitude of differential methylation or gene expression, our prioritization strategy integrated concordant DNA methylation and gene expression alterations together with pathway enrichment, biological relevance, previous evidence from the literature, and translational applicability. This biologically informed framework identified 51 candidate genes, from which *EPHA5* was prioritized for comprehensive independent validation. Importantly, *EPHA5* was not selected because it represented the most statistically significant alteration, but because it fulfilled multiple complementary criteria supporting its biological relevance and translational potential. This systematic approach provides a framework for biomarker prioritization that extends beyond statistical ranking alone.

The rationale for prioritizing *EPHA5* was further supported by accumulating evidence from multiple epithelial malignancies (34, 35, 37–42). Previous studies have consistently demonstrated promoter hypermethylation accompanied by reduced *EPHA5* expression in breast (34) and prostate cancers (35), with restoration of gene expression following pharmacological demethylation, supporting epigenetic regulation of this receptor. In addition, reduced EPHA5 protein expression has been associated with aggressive clinicopathological characteristics and poor prognosis in colorectal (38), renal (41), breast (34), bladder (40), prostate (35), and ovarian (37) cancers, while functional studies have demonstrated that EPHA5 depletion promotes cell migration and invasion in esophageal cancer models (43). Furthermore, methylated *EPHA5* detected in peripheral blood has been associated with an increased risk of gastric cancer (39), highlighting its potential translational applicability as a non-invasive biomarker. Collectively, these findings indicate that *EPHA5* represents a recurrent target of epigenetic dysregulation across multiple epithelial malignancies rather than a cancer-specific event. However, despite this growing body of evidence, the epigenetic and diagnostic significance of *EPHA5* in OSCC has remained largely unexplored, thereby supporting its selection for independent validation in the present study. Consistent with these observations, *EPHA5* promoter hypermethylation was detected in 66.7% of OSCC tumors in the present cohort, providing independent validation that *EPHA5* epigenetic dysregulation is also a frequent event in OSCC.

EPHA5 belongs to the Eph receptor tyrosine kinase family, the largest subgroup of receptor tyrosine kinases, which plays fundamental roles in embryonic development, tissue architecture, cell adhesion, migration and intercellular communication through bidirectional Eph/ephrin signaling (29, 44). Although originally recognized for their role in axon guidance, Eph receptors are now known to participate in multiple biological processes relevant to cancer, including cell proliferation, migration, invasion, cell adhesion and cytoskeletal remodeling (32, 36). Unlike many classical receptor tyrosine kinases that primarily promote mitogenic signaling, Eph receptors exhibit context-dependent functions and may act either as tumor promoters or tumor suppressors depending on tissue type, ligand availability, and downstream signaling networks. This biological complexity has been demonstrated across multiple malignancies, where different Eph family members exert distinct, and sometimes opposing, effects on tumor initiation and progression (32, 42, 45).

Consistent with these biological functions, pathway enrichment and protein-protein interaction analyses performed in the present study positioned EPHA5 within the axon guidance signaling pathway and predicted interactions with several proteins involved in Eph/ephrin signaling and intracellular signaling cascades (42, 45). Within this network, EPHA5 was linked to signaling molecules involved in MAPK signaling and cellular architecture, providing a biologically plausible framework through which epigenetic dysregulation of *EPHA5* may contribute to tumor progression (36). Nevertheless, because the present study did not include functional experiments, these pathway associations should be regarded as supportive biological context rather than direct mechanistic evidence.

The present findings are consistent with the putative tumor-suppressive functions that have previously been described for *EPHA5* in several epithelial malignancies such as gastric, breast, and prostate cancers (33–35). In the present cohort, *EPHA5* promoter hypermethylation was accompanied by reduced gene expression in a subset of OSCCs, supporting a potential association between promoter methylation and transcriptional downregulation during oral carcinogenesis. However, concordant methylation-associated transcriptional downregulation was observed only in a subset of tumors, suggesting that promoter methylation alone is unlikely to account for the heterogeneous *EPHA5* expression patterns observed in OSCC and that additional regulatory mechanisms may also contribute to its transcriptional regulation. Although the present study was not designed to establish a causal relationship between promoter methylation and transcriptional regulation, the concordant methylation-expression pattern observed in both the discovery and validation cohorts, together with previous experimental evidence from other tumor types, supports the biological plausibility of *EPHA5* epigenetic dysregulation in OSCC.

The present study extends previous observations by independently validating *EPHA5* promoter methylation and gene expression in a clinically well-characterized cohort comprising patients with OSCC, OPML and healthy controls using paired tissue specimens together with matched saliva and serum samples. This study design enabled not only independent verification of the genome-wide discovery findings but also assessment of the diagnostic specificity and translational applicability of *EPHA5* across different stages of oral carcinogenesis. *EPHA5* promoter methylation was frequently detected in OSCC tissues and remained detectable in saliva, indicating that methylated *EPHA5* DNA can be identified in oral fluids. Importantly, promoter methylation was also observed in more than half of OPML lesions. Although salivary methylation is unlikely to directly reflect functional gene silencing within tumor cells, its detection in saliva supports the potential diagnostic, rather than mechanistic, utility of *EPHA5* as a candidate non-invasive epigenetic biomarker. Because DNA was extracted from the salivary cell pellet, the detected methylation signal may originate from multiple cellular sources within the oral cavity, including exfoliated epithelial cells and inflammatory cells. Consequently, the present study cannot determine the precise cellular origin of salivary methylated DNA.

Diagnostic analyses demonstrated that *EPHA5* promoter methylation showed moderate discriminatory performance for distinguishing OSCC from healthy controls but did not reliably discriminate OSCC from OPML. In contrast, serum *EPHA5* methylation demonstrated poor diagnostic performance, indicating limited utility as a non-invasive biomarker in the present cohort. Furthermore, because salivary methylation was significantly associated with sex in the present cohort, the observed diagnostic performance should be interpreted with appropriate caution. The relatively high frequency of *EPHA5* promoter methylation observed in OPML, together with its limited ability to distinguish OSCC from OPML, is consistent with the possibility that this epigenetic alteration may arise before the development of invasive disease and persist throughout multiple stages of oral carcinogenesis. Consequently, *EPHA5* may have greater utility for risk stratification and longitudinal surveillance than as a stand-alone biomarker for differentiating OSCC from premalignant lesions. However, because the present study employed a cross-sectional design, it was not possible to determine whether *EPHA5*-methylated OPML lesions subsequently progressed to OSCC. Therefore, prospective longitudinal studies will be required to clarify the temporal relationship between *EPHA5* promoter methylation and malignant transformation. Although most associations between *EPHA5* methylation or expression and the evaluated clinicopathological variables were not statistically significant, reduced *EPHA5* expression showed a trend toward an association with lymph node metastasis in logistic regression analyses While this finding requires validation in larger independent cohorts, it suggests a potential association between reduced *EPHA5* expression and a more aggressive disease phenotype.

Epigenetic alterations have received considerable attention in OSCC because aberrant DNA methylation represents one of the most extensively investigated molecular alterations during oral carcinogenesis (6, 46, 47). Over the past two decades, numerous promoter methylation biomarkers have been proposed for the diagnosis, risk assessment and non-invasive detection of OSCC and HNSCC, including mismatch repair genes (48), *p16INK4a* (12), *KIF1A*, *EDNRB* (49, 50), *GNG7* (51), *NOL4* (52), *NID2*, *HOXA9* (53), as well as more recently *GABRB2* (54). Collectively, these studies highlight both the clinical potential of promoter methylation biomarkers and the marked molecular heterogeneity of OSCC, emphasizing the importance of systematic candidate prioritization.

Unlike most previous studies that focused on validating predefined candidate genes or methylation panels (49, 50, 53), the present study integrated genome-wide DNA methylation and transcriptomic profiling with pathway enrichment, protein interaction analysis, biological prioritization and independent clinical validation. Building on this integrated discovery strategy, previous genome-wide methylation studies have also identified *EPHA5* among differentially methylated or diagnostically relevant genes (55–57). Notably, to our knowledge, *EPHA5* was the only candidate consistently identified across these independent genome-wide methylation studies, supporting its reproducibility as an epigenetically altered target in OSCC. Although *EPHA5* demonstrated promising performance as an individual biomarker, incorporation into future multimarker epigenetic panels may further improve diagnostic accuracy. Exploratory ROC analyses further suggested that salivary *EPHA5* methylation may provide additional value for disease monitoring and risk stratification, although these findings require confirmation in larger independent cohorts. Because the present study identified a panel of candidate epigenetic biomarkers through an integrated genome-wide discovery strategy, future studies evaluating combinations of *EPHA5* with other candidate genes identified through the present discovery pipeline or with previously validated methylation biomarkers may further improve diagnostic accuracy and clinical applicability.

Several limitations of the present study should be acknowledged. First, the validation cohort was relatively small and derived from a single institution, which may have limited the statistical power of subgroup analyses. Second, functional experiments investigating the biological consequences of *EPHA5* promoter hypermethylation were beyond the scope of the present study; therefore, the observed associations should not be interpreted as evidence of a causal relationship between promoter methylation and transcriptional regulation. Third, the findings were not validated in an independent external cohort. Fourth, HPV status was unavailable for the analyzed samples, as HPV assessment was not incorporated into the original study design, which specifically focused on primary oral cavity OSCC rather than HPV-associated oropharyngeal squamous cell carcinoma. Although current CAP and ASCO guidelines do not recommend routine HPV testing for non-oropharyngeal squamous cell carcinomas, including oral cavity tumors, the potential contribution of HPV to a subset of OSCCs cannot be completely excluded (58, 59). Consistent with this, the biological role of HPV in OSCC remains controversial, and its etiological significance has not been clearly established (60). Consequently, larger multicenter studies integrating functional validation, external clinical validation, and HPV assessment will be necessary before *EPHA5* can be considered for routine clinical implementation.

Overall, the present study demonstrates that integrating genome-wide DNA methylation and transcriptomic profiling with biological prioritization and independent clinical validation provides an effective strategy for identifying clinically relevant epigenetic biomarkers. Within this framework, *EPHA5* emerged as a biologically and translationally relevant candidate biomarker whose promoter hypermethylation was consistently detected in both tumor tissue and saliva, supporting its potential for non-invasive OSCC detection. Further multicenter studies incorporating larger independent cohorts, functional validation, and multimarker epigenetic panels will be important to determine the clinical utility of *EPHA5* for oral cancer risk assessment, disease monitoring and clinical implementation. In addition, future studies evaluating other candidates identified through the present discovery pipeline may further refine multimarker epigenetic panels for OSCC.

## Data Availability

The genome-wide DNA methylation and gene expression datasets generated in this study are publicly available in the NCBI Gene Expression Omnibus (GEO) under accession numbers GSE208065 (DNA methylation), GSE208067 (gene expression), and GSE208068 (SuperSeries).

## References

[1] Sadeghzade A Jafarian A Davodpour AH Pouresmaeliyan Roumani M Mohammadikhah M Qutaiba Badraldeen S . Oral squamous cell carcinoma pharmacological treatment; a long non-coding RNAs (long ncRNAs) story. Int J Mol Cell Med. (2025) 14:736–52. doi: 10.22088/IJMCM.BUMS.14.2.736 PMC12321307 40765751

[2] Coppini M Seminara G Mauceri R Di Fede O La Mantia G Mauceri N . Field cancerization in women without conventional risk factors: insights from a case-cohort study. Front Oral Health. (2025) 6:1653210. doi: 10.3389/froh.2025.1653210 40895779 PMC12391006

[3] Montero PH Patel SG . Cancer of the oral cavity. Surg Oncol Clin N Am. (2015) 24:491–508. doi: 10.1016/j.soc.2015.03.006 PMC5018209 25979396

[4] Solanki Jitender GS Hiremath RV Yadav O Khan M . Screening for oral cancer. J Exp Ther Oncol. (2016) 11:303–7. 27849341

[5] Gupta I Badrzadeh F Tsentalovich Y Gaykalova DA . Connecting the dots: investigating the link between environmental, genetic, and epigenetic influences in metabolomic alterations in oral squamous cell carcinoma. J Exp Clin Cancer Res. (2024) 43:239. doi: 10.1186/s13046-024-03141-5 39169426 PMC11337877

[6] Vatsa PP Jindal Y Bhadwalkar J Chamoli A Upadhyay V Mandoli A . Role of epigenetics in OSCC: an understanding above genetics. Med Oncol. (2023) 40:122. doi: 10.1007/s12032-023-01992-0 36941511

[7] Wang S Yang M Li R Bai J . Current advances in noninvasive methods for the diagnosis of oral squamous cell carcinoma: a review. Eur J Med Res. (2023) 28:53. doi: 10.1186/s40001-022-00916-4 36707844 PMC9880940

[8] Gupta S Singh B Abhishek R Gupta S Sachan M . The emerging role of liquid biopsy in oral squamous cell carcinoma detection: advantages and challenges. Expert Rev Mol Diagn. (2024) 24:311–31. doi: 10.1080/14737159.2024.2340997 38607339

[9] Ibrahim J Peeters M Van Camp G Op de Beeck K . Methylation biomarkers for early cancer detection and diagnosis: current and future perspectives. Eur J Cancer. (2023) 178:91–113. doi: 10.1016/j.ejca.2022.10.015 36427394

[10] Li LC Dahiya R . MethPrimer: designing primers for methylation PCRs. Bioinformatics. (2002) 18:1427–31. doi: 10.1093/bioinformatics/18.11.1427 12424112

[11] Ye J Coulouris G Zaretskaya I Cutcutache I Rozen S Madden TL . Primer-BLAST: a tool to design target-specific primers for polymerase chain reaction. BMC Bioinf. (2012) 13:134. doi: 10.1186/1471-2105-13-134 22708584 PMC3412702

[12] Demokan S Chuang A Suoglu Y Ulusan M Yalniz Z Califano JA . Promoter methylation and loss of p16(INK4a) gene expression in head and neck cancer. Head Neck. (2012) 34:1470–5. doi: 10.1002/hed.21949 22106032

[13] Livak KJ Schmittgen TD . Analysis of relative gene expression data using real-time quantitative PCR and the 2(-Delta Delta C(T)) method. Methods. (2001) 25:402–8. doi: 10.1006/meth.2001.1262 11846609

[14] Morris TJ Butcher LM Feber A Teschendorff AE Chakravarthy AR Wojdacz TK . ChAMP: 450k chip analysis methylation pipeline. Bioinformatics. (2014) 30:428–30. doi: 10.1093/bioinformatics/btt684 24336642 PMC3904520

[15] Team RC . R: A Language and Environment for Statistical Computing. Vienna: R Foundation for Statistical Computing (2021).

[16] Aryee MJ Jaffe AE Corrada-Bravo H Ladd-Acosta C Feinberg AP Hansen KD . Minfi: a flexible and comprehensive Bioconductor package for the analysis of Infinium DNA methylation microarrays. Bioinformatics. (2014) 30:1363–9. doi: 10.1093/bioinformatics/btu049 24478339 PMC4016708

[17] Fortin JP Triche TJ Jr. Hansen KD . Preprocessing, normalization and integration of the Illumina HumanMethylationEPIC array with minfi. Bioinformatics. (2017) 33:558–60. doi: 10.1093/bioinformatics/btw691 28035024 PMC5408810

[18] Zhou W Laird PW Shen H . Comprehensive characterization, annotation and innovative use of Infinium DNA methylation BeadChip probes. Nucleic Acids Res. (2017) 45:e22. doi: 10.1093/nar/gkw967 27924034 PMC5389466

[19] Nordlund J Backlin CL Wahlberg P Busche S Berglund EC Eloranta ML . Genome-wide signatures of differential DNA methylation in pediatric acute lymphoblastic leukemia. Genome Biol. (2013) 14:r105. doi: 10.1186/gb-2013-14-9-r105 24063430 PMC4014804

[20] Teschendorff AE Marabita F Lechner M Bartlett T Tegner J Gomez-Cabrero D . A beta-mixture quantile normalization method for correcting probe design bias in Illumina Infinium 450 k DNA methylation data. Bioinformatics. (2013) 29:189–96. doi: 10.1093/bioinformatics/bts680 23175756 PMC3546795

[21] Teschendorff AE Menon U Gentry-Maharaj A Ramus SJ Gayther SA Apostolidou S . An epigenetic signature in peripheral blood predicts active ovarian cancer. PloS One. (2009) 4:e8274. doi: 10.1371/journal.pone.0008274 20019873 PMC2793425

[22] Johnson WE Li C Rabinovic A . Adjusting batch effects in microarray expression data using empirical Bayes methods. Biostatistics. (2007) 8:118–27. doi: 10.1093/biostatistics/kxj037 16632515

[23] Leek JT Johnson WE Parker HS Jaffe AE Storey JD . The sva package for removing batch effects and other unwanted variation in high-throughput experiments. Bioinformatics. (2012) 28:882–3. doi: 10.1093/bioinformatics/bts034 22257669 PMC3307112

[24] Butcher LM Beck S . Probe Lasso: a novel method to rope in differentially methylated regions with 450K DNA methylation data. Methods. (2015) 72:21–8. doi: 10.1016/j.ymeth.2014.10.036 25461817 PMC4304833

[25] Du P Kibbe WA Lin SM . lumi: a pipeline for processing Illumina microarray. Bioinformatics. (2008) 24:1547–8. doi: 10.1093/bioinformatics/btn224 18467348

[26] Smyth GK . limma: linear models for microarray data. In: Gentleman R Carey VJ Huber W Irizarry RA Dudoit S , editors. Bioinformatics and Computational Biology Solutions Using R and Bioconductor. Statistics for Biology and Health. Springer, New York, NY (2005).

[27] Gu Z . Complex heatmap visualization. Imeta. (2022) 1:e43. doi: 10.1002/imt2.43 38868715 PMC10989952

[28] Ulgen E Ozisik O Sezerman OU . pathfindR: an R package for comprehensive identification of enriched pathways in omics data through active subnetworks. Front Genet. (2019) 10:858. doi: 10.3389/fgene.2019.00858 31608109 PMC6773876

[29] Kanehisa M Goto S . KEGG: kyoto encyclopedia of genes and genomes. Nucleic Acids Res. (2000) 28:27–30. doi: 10.1093/nar/28.1.27 10592173 PMC102409

[30] IBM Corp . IBM SPSS Statistics for Windows, Version 31.0. Armonk, NY: IBM Corp. (2013).

[31] Vogelstein B Papadopoulos N Velculescu VE Zhou S Diaz LA Jr. Kinzler KW . Cancer genome landscapes. Science. (2013) 339:1546–58. doi: 10.1126/science.1235122 23539594 PMC3749880

[32] Anderton M van der Meulen E Blumenthal MJ Schafer G . The role of the Eph receptor family in tumorigenesis. Cancers (Basel). (2021) 13. doi: 10.3390/cancers13020206 33430066 PMC7826860

[33] Eyvazi S Khamaneh AM Tarhriz V Bandehpour M Hejazi MS Sadat ATE . CpG islands methylation analysis of CDH11, EphA5, and HS3ST2 genes in gastric adenocarcinoma patients. J Gastrointest Cancer. (2020) 51:579–83. doi: 10.1007/s12029-019-00290-1 31407253

[34] Fu DY Wang ZM Wang BL Chen L Yang WT Shen ZZ . Frequent epigenetic inactivation of the receptor tyrosine kinase EphA5 by promoter methylation in human breast cancer. Hum Pathol. (2010) 41:48–58. doi: 10.1016/j.humpath.2009.06.007 19733895

[35] Li S Zhu Y Ma C Qiu Z Zhang X Kang Z . Downregulation of EphA5 by promoter methylation in human prostate cancer. BMC Cancer. (2015) 15:18. doi: 10.1186/s12885-015-1025-3 25609195 PMC4307617

[36] Miao H Wei BR Peehl DM Li Q Alexandrou T Schelling JR . Activation of EphA receptor tyrosine kinase inhibits the Ras/MAPK pathway. Nat Cell Biol. (2001) 3:527–30. doi: 10.1038/35074604 11331884

[37] Chen X Wang X Wei X Wang J . EphA5 protein, a potential marker for distinguishing histological grade and prognosis in ovarian serous carcinoma. J Ovarian Res. (2016) 9:83. doi: 10.1186/s13048-016-0292-1 27887627 PMC5123222

[38] Gu S Feng J Jin Q Wang W Zhang S . Reduced expression of EphA5 is associated with lymph node metastasis, advanced TNM stage, and poor prognosis in colorectal carcinoma. Histol Histopathol. (2017) 32:491–7. doi: 10.14670/HH-11-815 27651378

[39] Han X Liu T Zhai J Liu C Wang W Nie C . Association between EPHA5 methylation status in peripheral blood leukocytes and the risk and prognosis of gastric cancer. PeerJ. (2022) 10:e13774. doi: 10.7717/peerj.13774 36164608 PMC9508887

[40] Lopez JI Angulo JC Martin A Sanchez-Chapado M Gonzalez-Corpas A Colas B . A DNA hypermethylation profile reveals new potential biomarkers for the evaluation of prognosis in urothelial bladder cancer. APMIS. (2017) 125:787–96. doi: 10.1111/apm.12719 28586118

[41] Wang X Xu H Wu Z Chen X Wang J . Decreased expression of EphA5 is associated with Fuhrman nuclear grade and pathological tumour stage in ccRCC. Int J Exp Pathol. (2017) 98:34–9. doi: 10.1111/iep.12219 28421649 PMC5447857

[42] Zhang W Cao H Yang J Zhao J Liang Z Kang X . The identification and validation of EphA7 hypermethylation, a novel biomarker, in cervical cancer. BMC Cancer. (2022) 22:636. doi: 10.1186/s12885-022-09653-7 35681118 PMC9185921

[43] Zhang R Liu J Zhang W Hua L Qian LT Zhou SB . EphA5 knockdown enhances the invasion and migration ability of esophageal squamous cell carcinoma via epithelial-mesenchymal transition through activating Wnt/beta-catenin pathway. Cancer Cell Int. (2020) 20:20. doi: 10.1186/s12935-020-1101-x 31956298 PMC6958788

[44] Szklarczyk D Kirsch R Koutrouli M Nastou K Mehryary F Hachilif R . The STRING database in 2023: protein-protein association networks and functional enrichment analyses for any sequenced genome of interest. Nucleic Acids Res. (2023) 51:D638–46. doi: 10.1093/nar/gkac1000 36370105 PMC9825434

[45] Lavareze L Kimura TC Scarini JF de Lima-Souza RA Goncalves MWA Sales de Sa R . Advances and current concepts on Eph receptors and ephrins in upper digestive tract cancers. Front Oncol. (2024) 14:1520306. doi: 10.3389/fonc.2024.1520306 39839790 PMC11746119

[46] Agarwal N Jha AK . DNA hypermethylation of tumor suppressor genes among oral squamous cell carcinoma patients: a prominent diagnostic biomarker. Mol Biol Rep. (2024) 52:44. doi: 10.1007/s11033-024-10144-0 39644423

[47] Gonzalez-Ramirez I Garcia-Cuellar C Sanchez-Perez Y Granados-Garcia M . DNA methylation in oral squamous cell carcinoma: molecular mechanisms and clinical implications. Oral Dis. (2011) 17:771–8. doi: 10.1111/j.1601-0825.2011.01833.x 21781230

[48] Demokan S Suoglu Y Demir D Gozeler M Dalay N . Microsatellite instability and methylation of the DNA mismatch repair genes in head and neck cancer. Ann Oncol. (2006) 17:995–8. doi: 10.1093/annonc/mdl048 16569647

[49] Demokan S Chang X Chuang A Mydlarz WK Kaur J Huang P . KIF1A and EDNRB are differentially methylated in primary HNSCC and salivary rinses. Int J Cancer. (2010) 127:2351–9. doi: 10.1002/ijc.25248 20162572 PMC2946472

[50] Pattani KM Zhang Z Demokan S Glazer C Loyo M Goodman S . Endothelin receptor type B gene promoter hypermethylation in salivary rinses is independently associated with risk of oral cavity cancer and premalignancy. Cancer Prev Res (Phila). (2010) 3:1093–103. doi: 10.1158/1940-6207.capr-10-0115 20798208 PMC2945229

[51] Demokan S Chuang AY Chang X Khan T Smith IM Pattani KM . Identification of guanine nucleotide-binding protein gamma-7 as an epigenetically silenced gene in head and neck cancer by gene expression profiling. Int J Oncol. (2013) 42:1427–36. doi: 10.3892/ijo.2013.1808 23403885 PMC3981008

[52] Demokan S Chuang AY Pattani KM Sidransky D Koch W Califano JA . Validation of nucleolar protein 4 as a novel methylated tumor suppressor gene in head and neck cancer. Oncol Rep. (2014) 31:1014–20. doi: 10.3892/or.2013.2927 24337411 PMC3896520

[53] Guerrero-Preston R Soudry E Acero J Orera M Moreno-Lopez L Macia-Colon G . NID2 and HOXA9 promoter hypermethylation as biomarkers for prevention and early detection in oral cavity squamous cell carcinoma tissues and saliva. Cancer Prev Res (Phila). (2011) 4:1061–72. doi: 10.1158/1940-6207.capr-11-0006 21558411 PMC3131432

[54] Sen S Doruk C Ulusan M Olgac V Ak G Demokan S . Methylation-driven downregulation of GABRB2 in oral carcinoma's subtype: implications for early diagnosis and differentiation. BMC Oral Health. (2025) 25:1713. doi: 10.1186/s12903-025-06899-y 41174677 PMC12579410

[55] Jithesh PV Risk JM Schache AG Dhanda J Lane B Liloglou T . The epigenetic landscape of oral squamous cell carcinoma. Br J Cancer. (2013) 108:370–9. doi: 10.1038/bjc.2012.568 23287992 PMC3566828

[56] Li YF Hsiao YH Lai YH Chen YC Chen YJ Chou JL . DNA methylation profiles and biomarkers of oral squamous cell carcinoma. Epigenetics. (2015) 10:229–36. doi: 10.1080/15592294.2015.1006506 25612142 PMC4622594

[57] Viet CT Schmidt BL . Methylation array analysis of preoperative and postoperative saliva DNA in oral cancer patients. Cancer Epidemiol Biomarkers Prev. (2008) 17:3603–11. doi: 10.1158/1055-9965.epi-08-0507 19064577

[58] Fakhry C Lacchetti C Perez-Ordonez B . Human papillomavirus testing in head and neck carcinomas: ASCO clinical practice guideline endorsement summary of the CAP guideline. J Oncol Pract. (2018) 14:613–7. doi: 10.1200/jop.18.00433 30192693

[59] James S Lewis J B.B. Bishop JA Chernock RD Colasacco C Lacchetti C . Human papillomavirus testing in head and neck carcinomas. Arch Pathol Lab Med. (2018) 142:559–7. doi: 10.5858/arpa.2017-0286-CP 29251996

[60] Hubbers CU Akgul B . HPV and cancer of the oral cavity. Virulence. (2015) 6:244–8. doi: 10.1080/21505594.2014.999570 PMC4601238 25654476

