# Supplementary material for: Integrated genome-wide DNA methylation and transcriptomic analysis prioritizes EPHA5 as a candidate non-invasive epigenetic biomarker for oral squamous cell carcinoma
Source: Front Oncol. 2026 Oct 2;16:1932336. doi: 10.3389/fonc.2026.1932336 (PMC13671974; doi:10.3389/fonc.2026.1932336)
Supplement: Supplementary file 1 [file Image1.pdf]

(A)

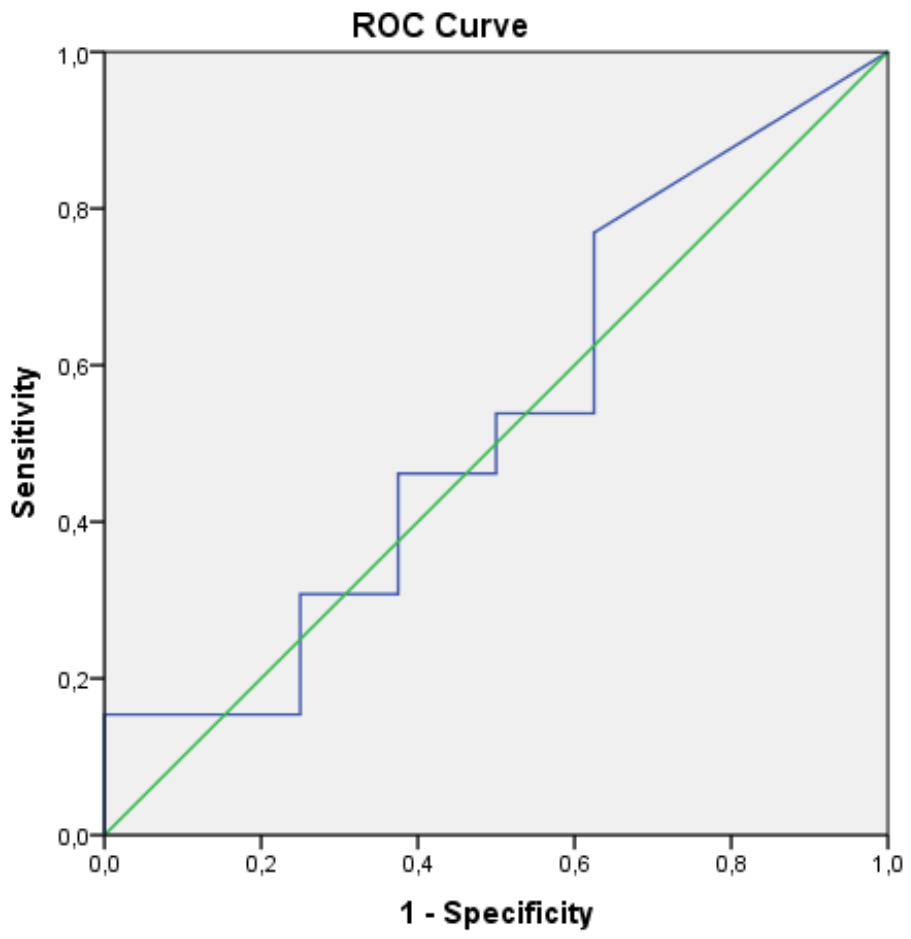
ROC Curve
Sensitivity
1 - Specificity
0,0
0,2
0,4
0,6
0,8
1,0

(B)

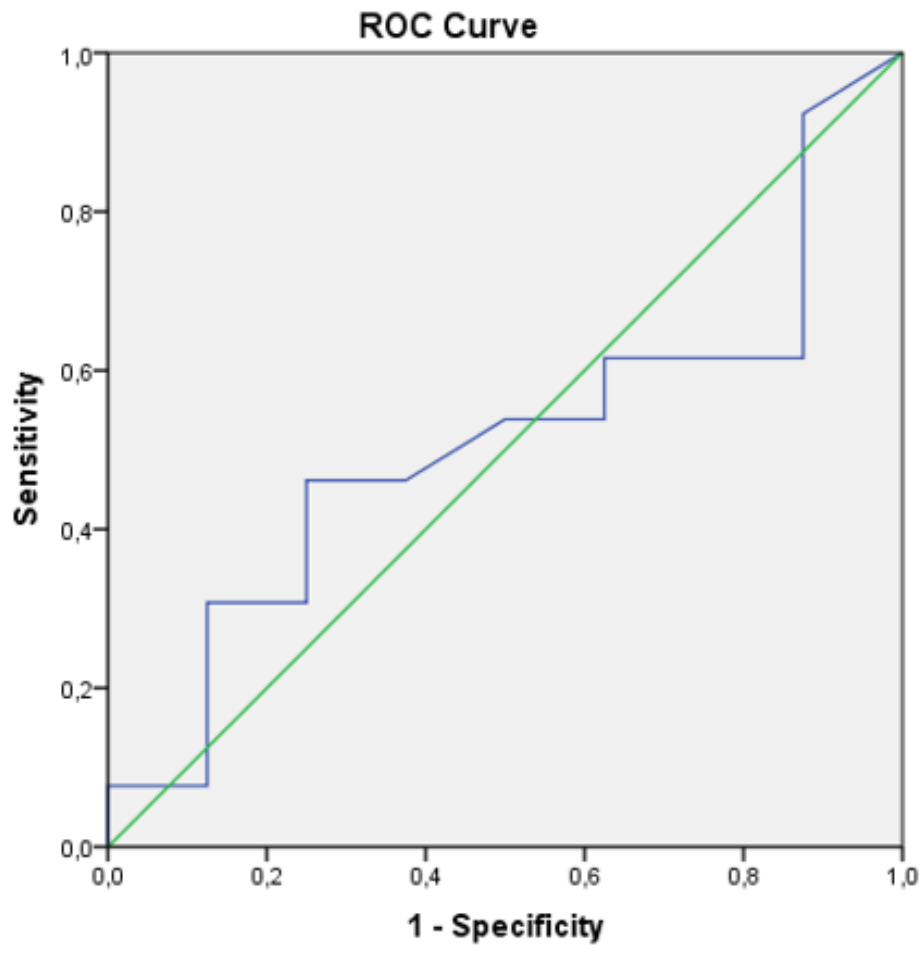
ROC Curve
Sensitivity
1 - Specificity
0,0
0,2
0,4
0,6
0,8
1,0

(C)

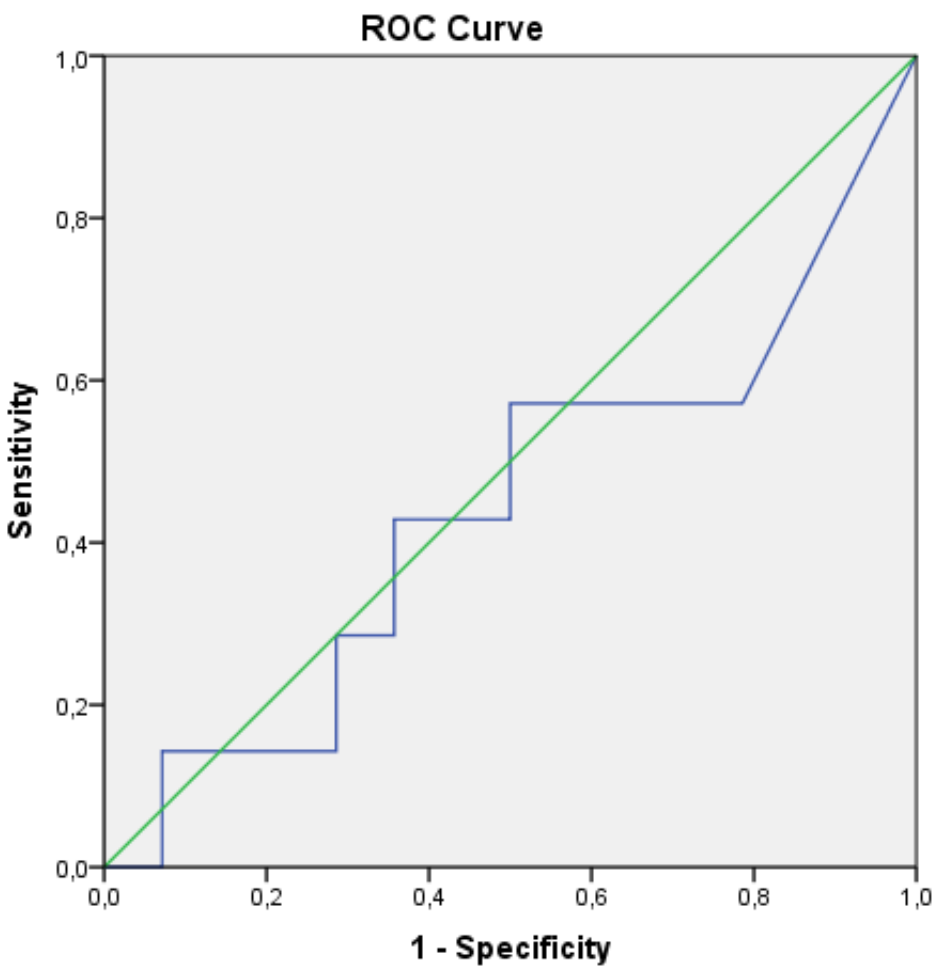
ROC Curve
Sensitivity
1 - Specificity
0,0
0,2
0,4
0,6
0,8
1,0

(D)

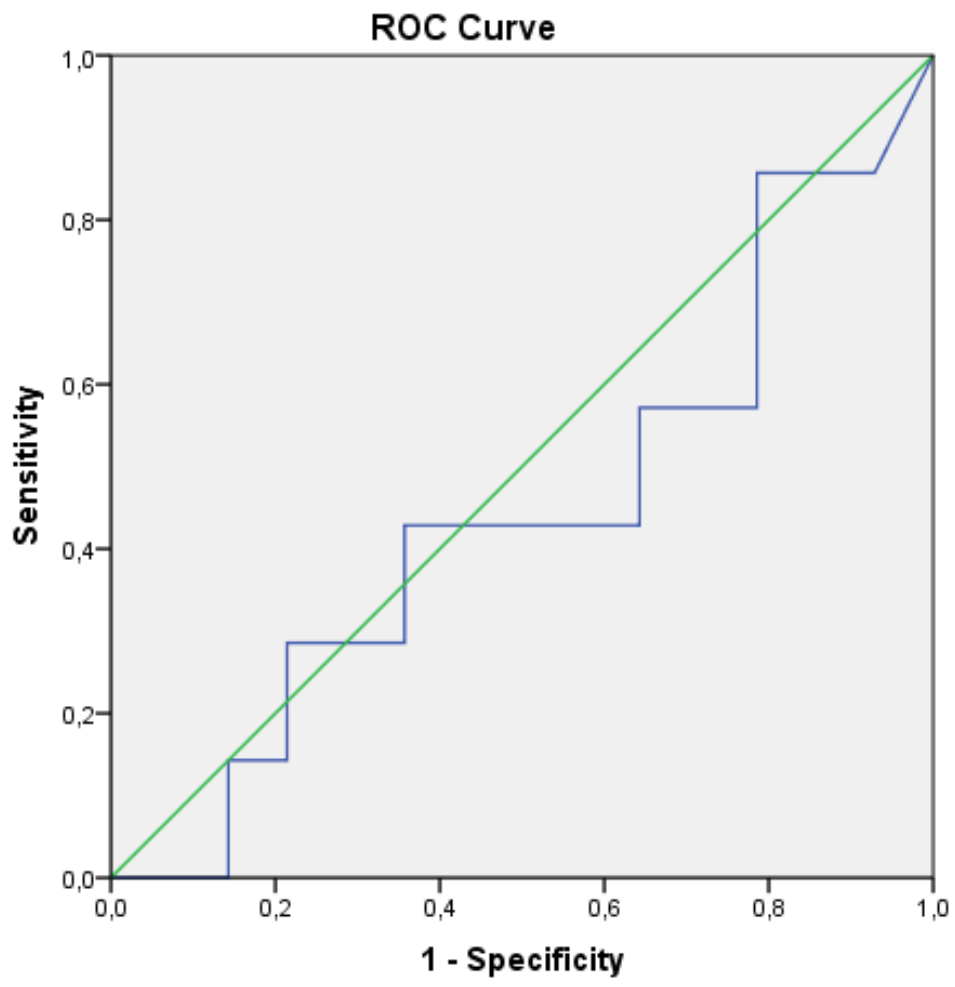
ROC Curve
Sensitivity
1 - Specificity
0,0
0,2
0,4
0,6
0,8
1,0

(E)

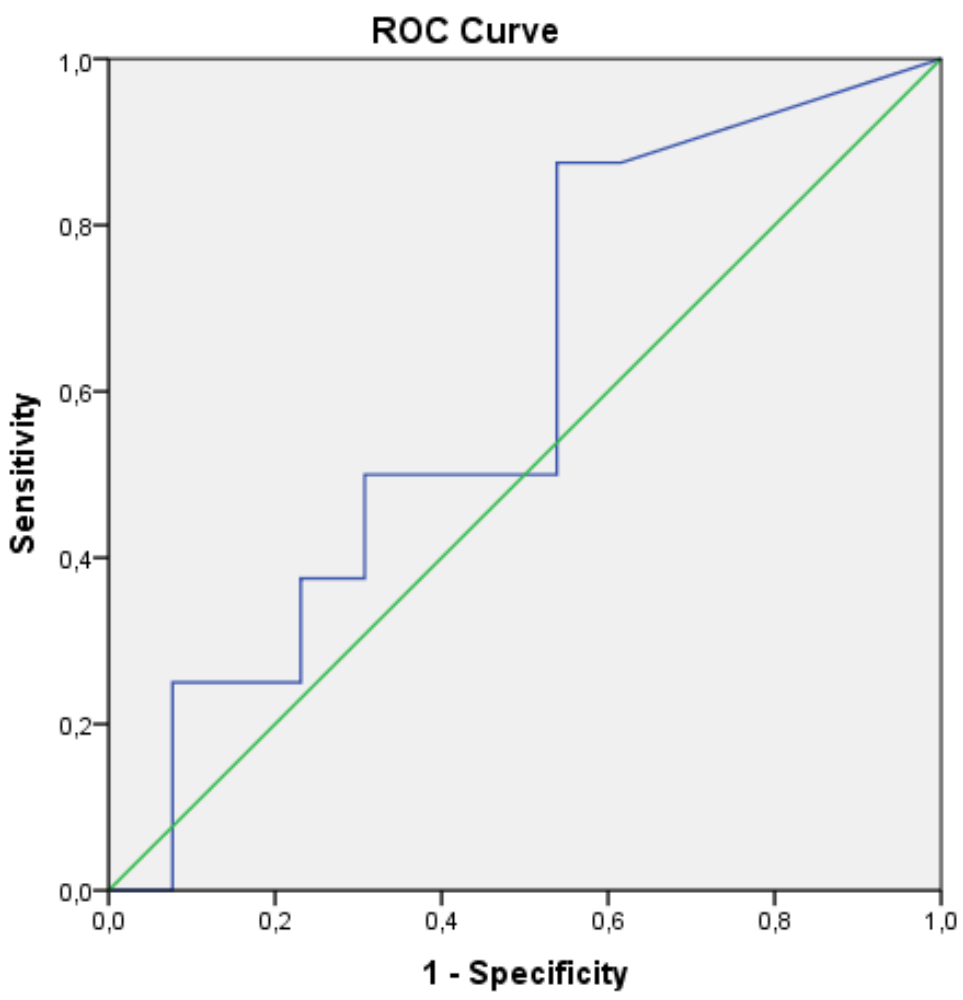
ROC Curve
Sensitivity
1 - Specificity
0,0
0,2
0,4
0,6
0,8
1,0

(F)

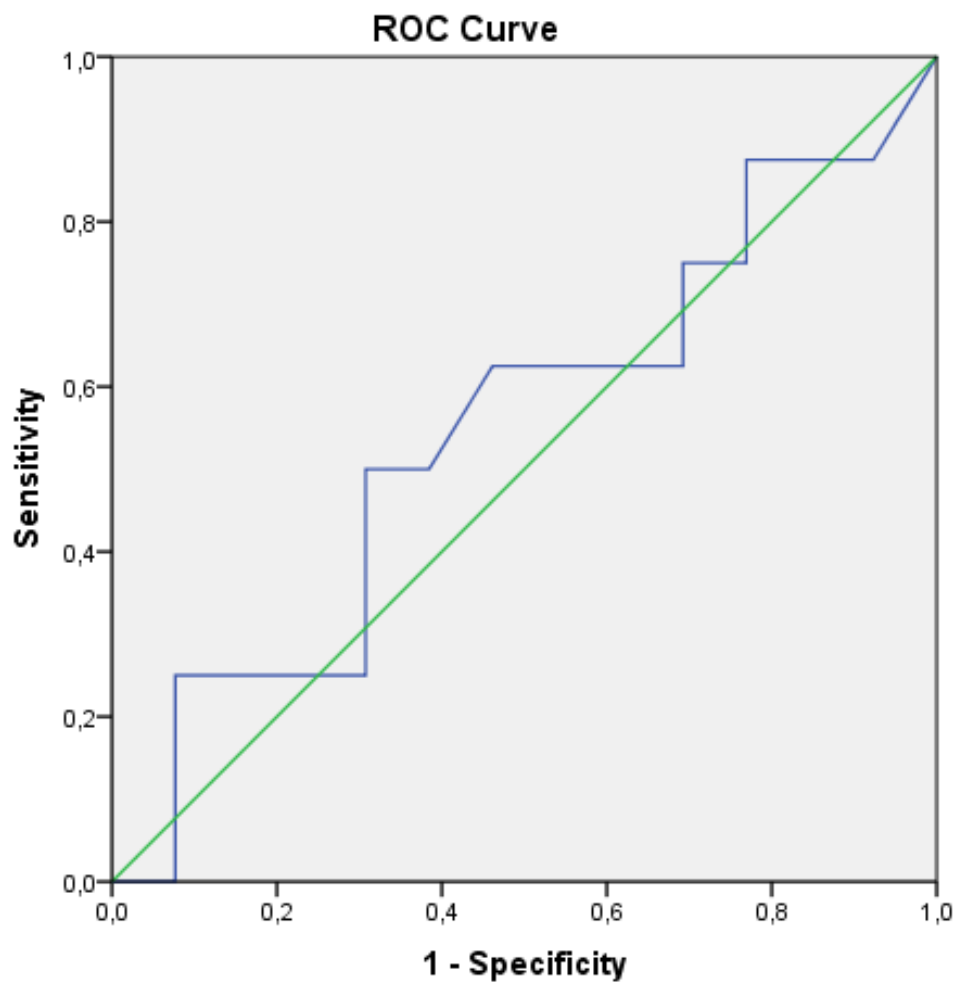
ROC Curve
Sensitivity
1 - Specificity
0,0
0,2
0,4
0,6
0,8
1,0

(G)

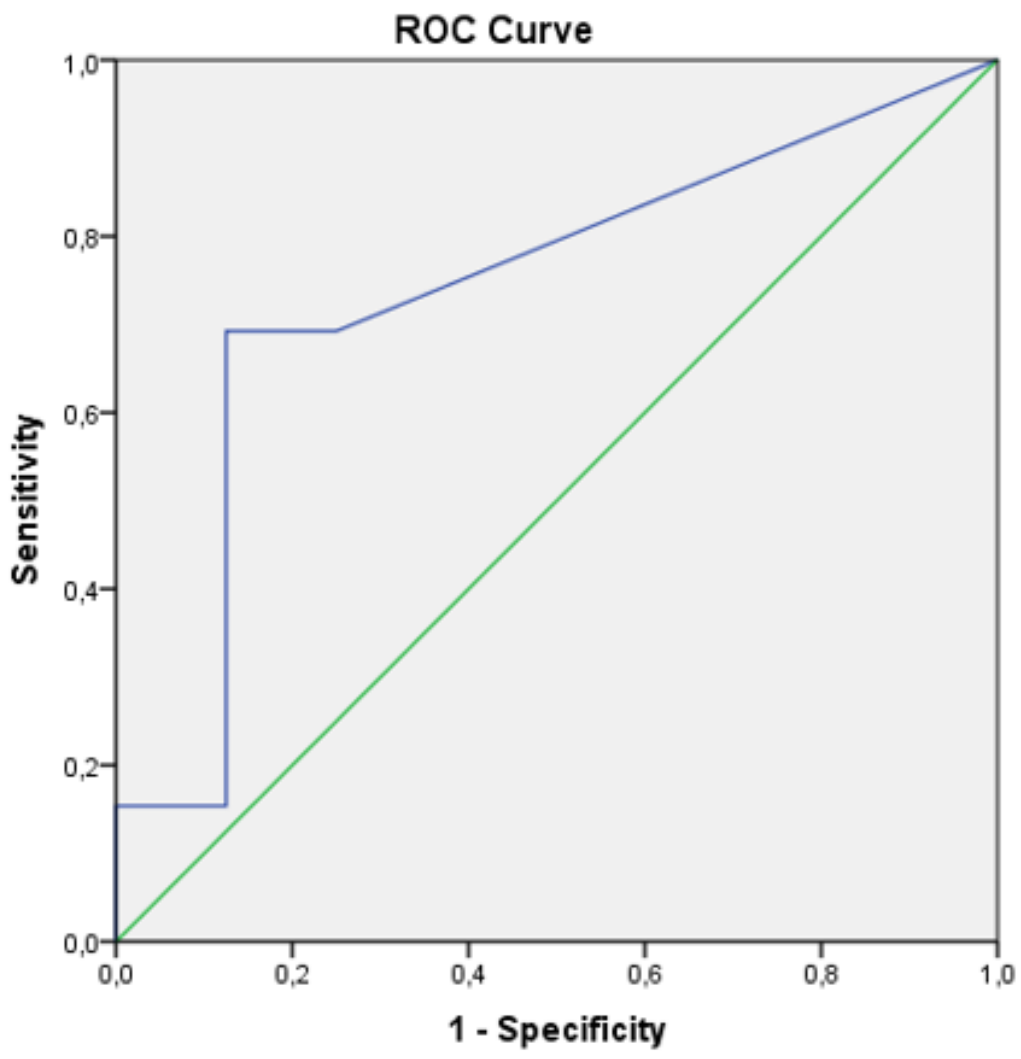


(H)

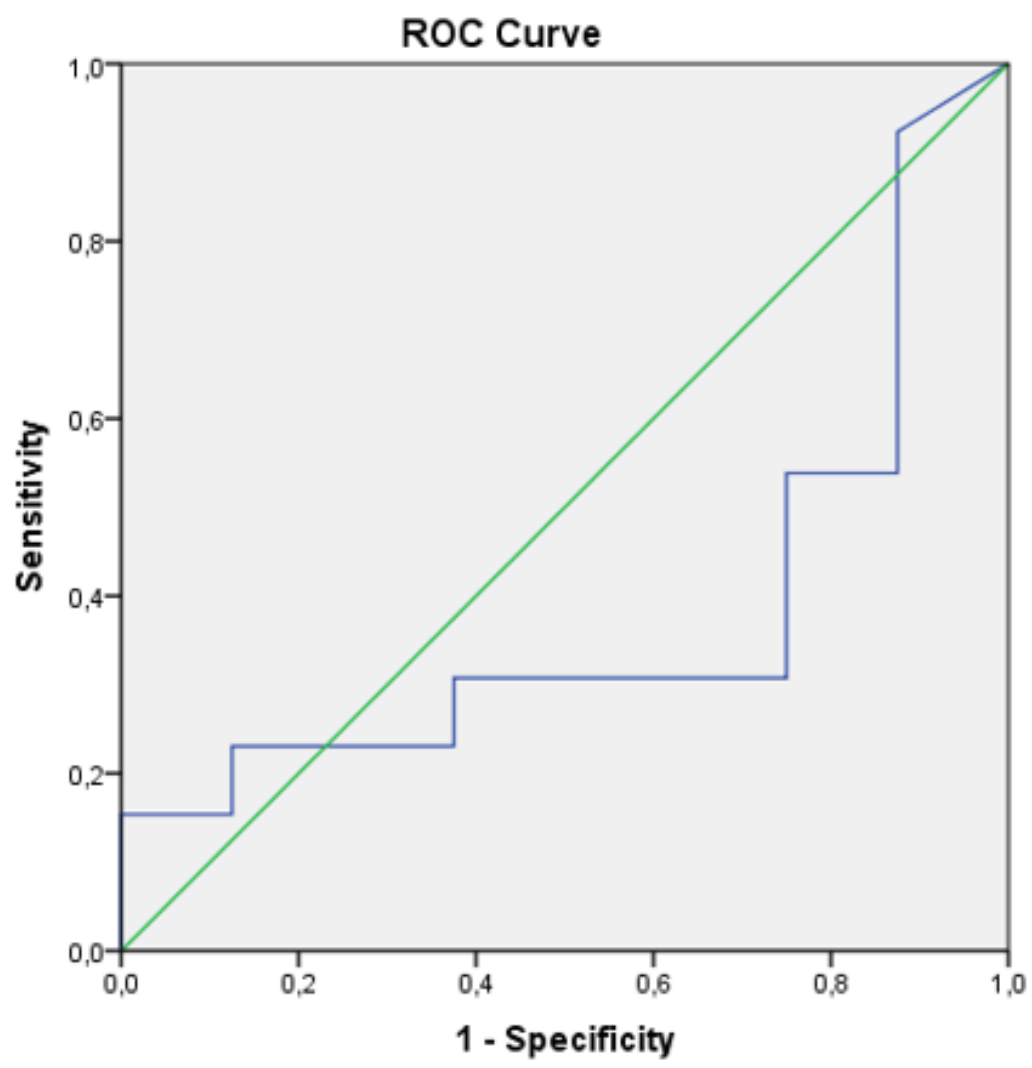


(I)

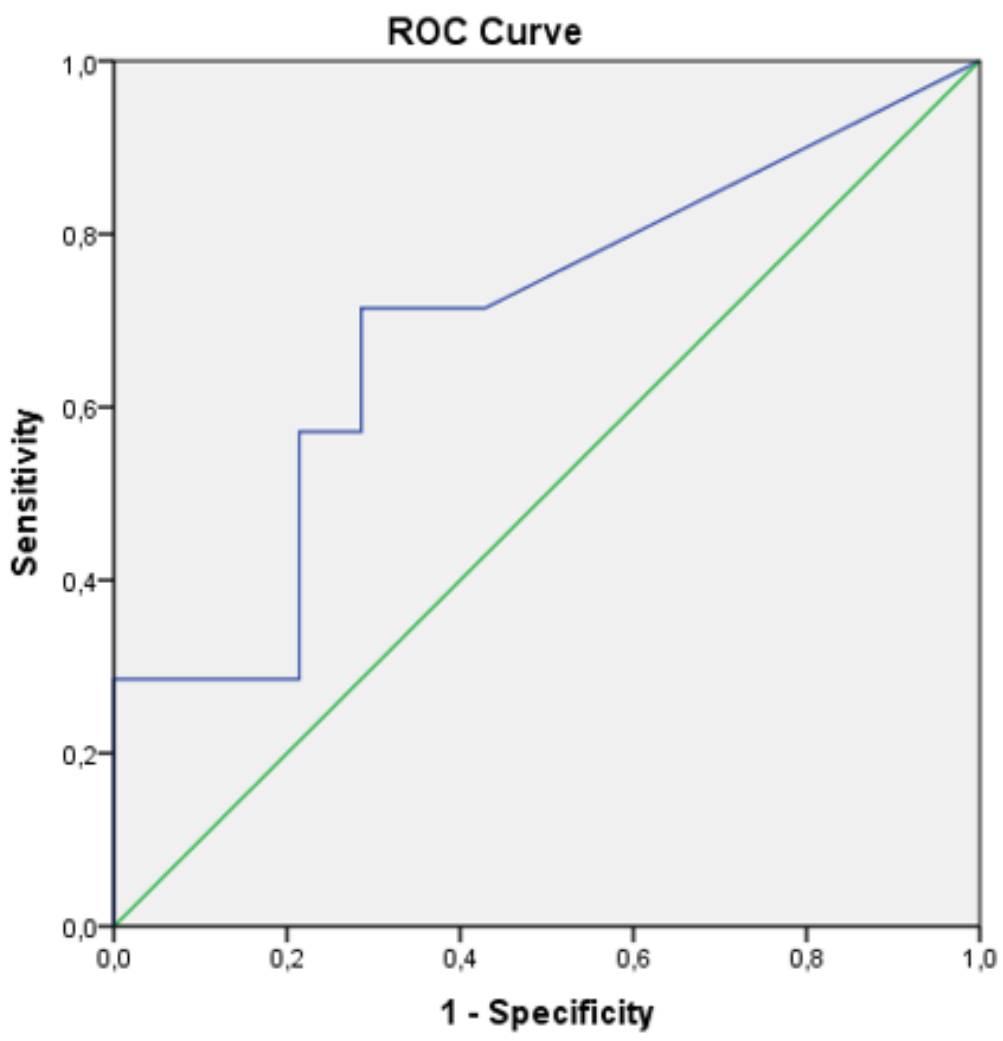


(J)

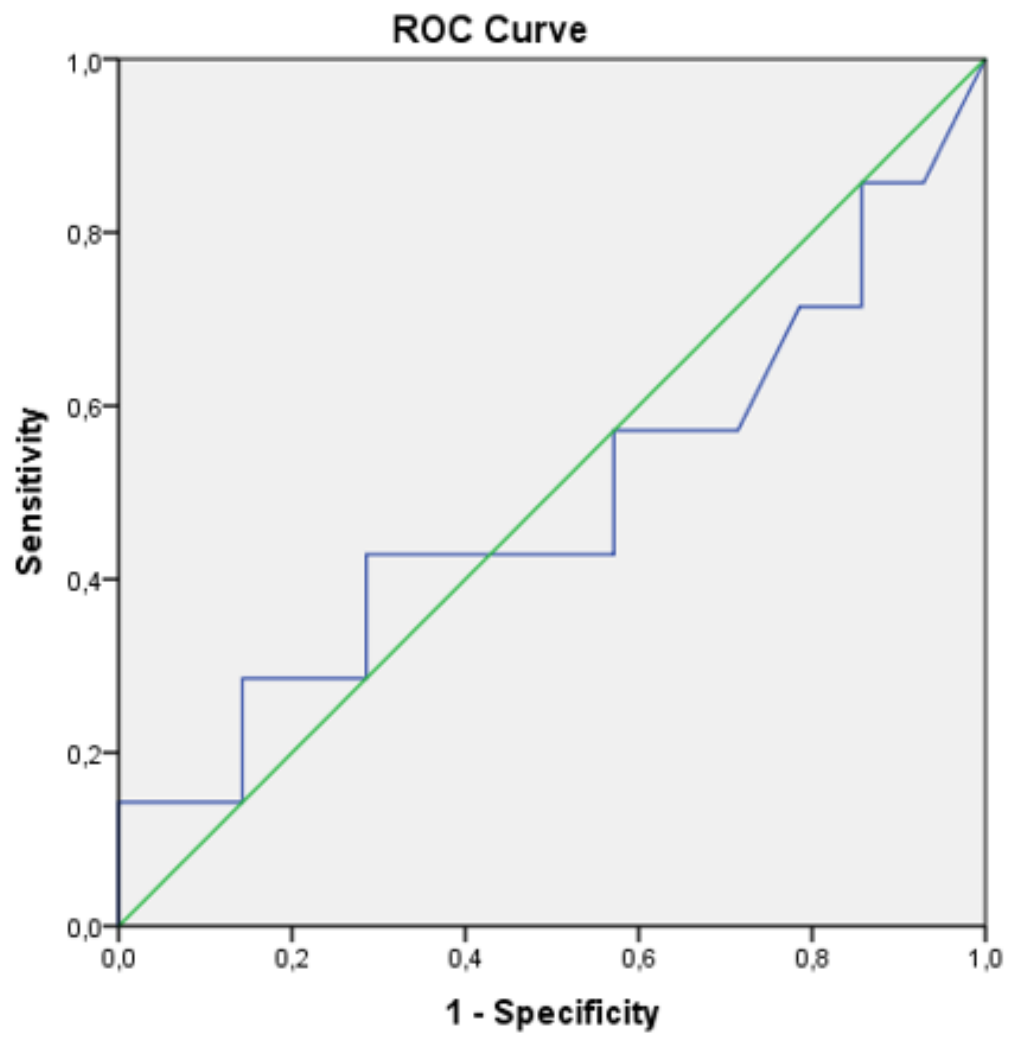


(K)

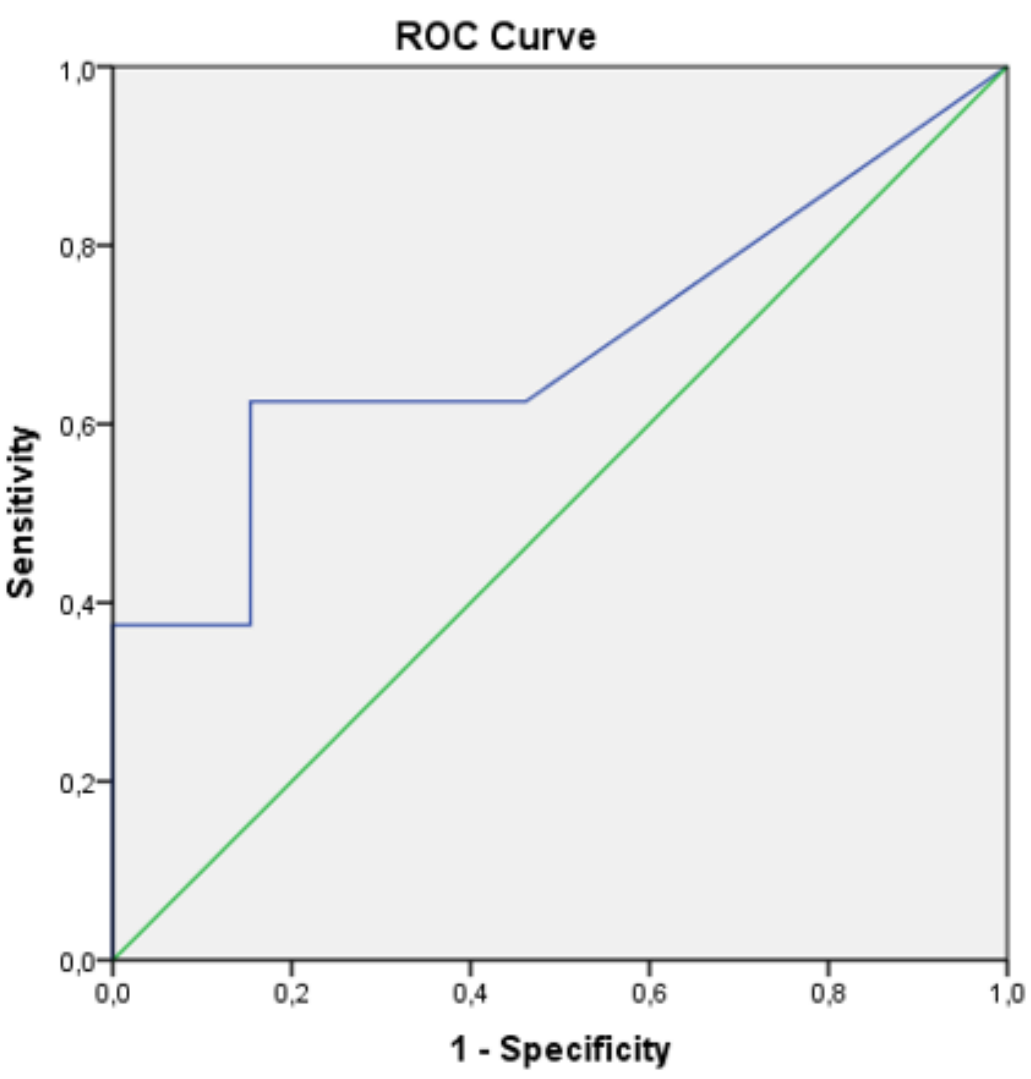


(L)

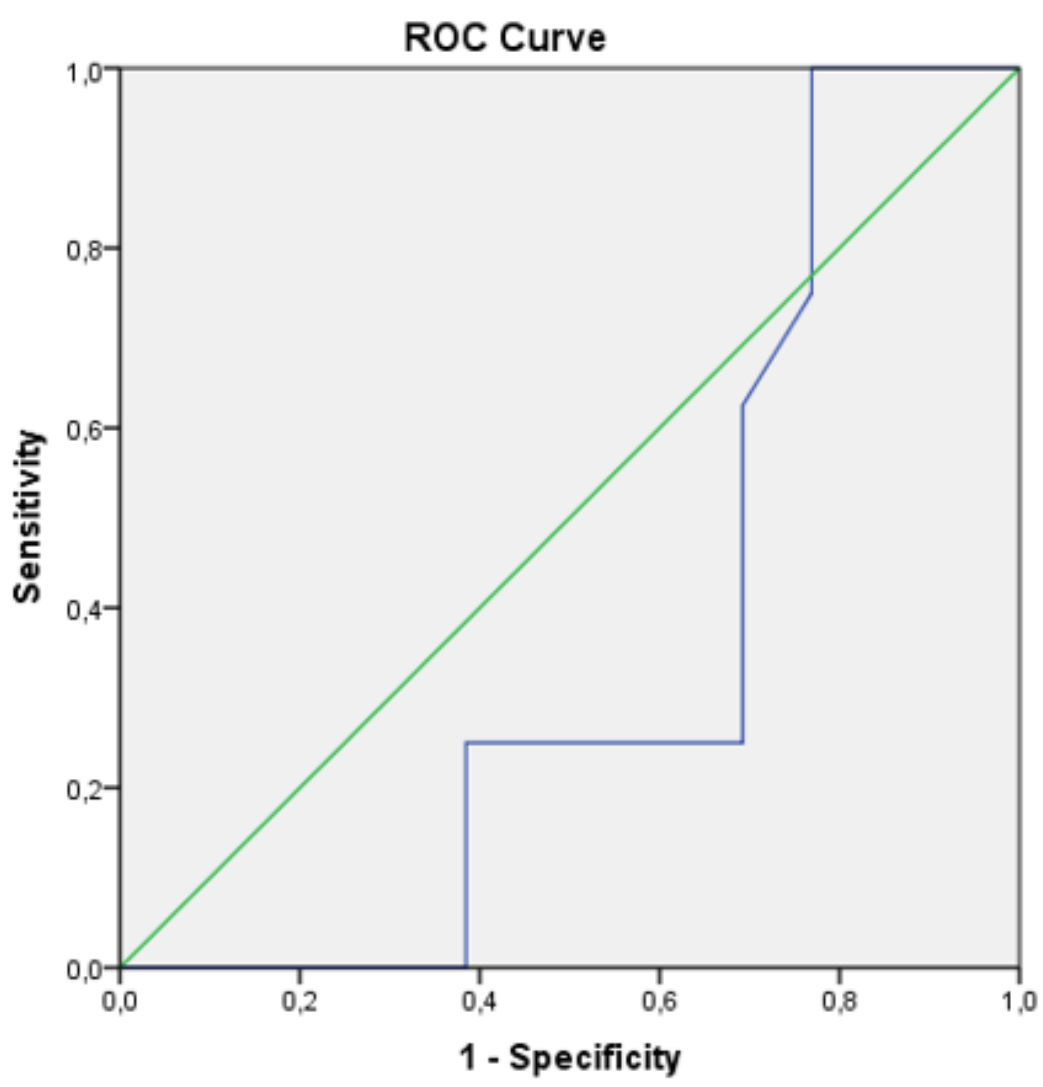


**Supplementary Figure S1**: Exploratory receiver operating characteristic (ROC) analyses of

*EPHA5* methylation and expression according to clinicopathological outcomes in OSCC. ROC curves showing the discriminatory performance of tumor methylation (A, C, E), tumor gene expression (B, D, F), salivary methylation (G, I, K), and salivary gene expression (H, J, L) for distinguishing early-stage versus advanced-stage disease (A, B, G, H), metastatic versus non-metastatic disease (C, D, I, J), and recurrent versus non-recurrent disease (E, F, K, L). None of the analyses reached statistical significance (all $p > 0.05$), although salivary methylation demonstrated borderline discriminatory performance for early-stage versus advanced-stage disease (AUC = 0.740, $p = 0.070$) and moderate but non-significant performance for metastatic status (AUC = 0.694, $p = 0.156$) and recurrence (AUC = 0.688, $p = 0.158$).
