# Supplementary material for: Integrated genome-wide DNA methylation and transcriptomic analysis prioritizes EPHA5 as a candidate non-invasive epigenetic biomarker for oral squamous cell carcinoma
Source: Front Oncol. 2026 Oct 2;16:1932336. doi: 10.3389/fonc.2026.1932336 (PMC13671974; doi:10.3389/fonc.2026.1932336)
Supplement: Supplementary file 2 [file Table5.pdf]

Supplementary Table S5. Univariate and multivariate logistic regression analyses in OSCC patients with expression loss

**Parameters**	**Univariate Analysis**		**Multivariate Analysis**	
	**OR (95%CI)**	**p****	**OR (95%CI)**	**p****
**Age (year)**	0.98(0.90-1.06	0.60	-	-
**Gender**	0.72 (0.12-4.21)	0.71	-	-
**Anatomical site**	1.58 (0.54-4.61)	0.41	-	-
**Smoking**	0.60 (0.10-3.72)	0.58	-	-
**Differentiation Degree**	1.44 (0.48-4.31)	0.51	-	-
**Early/Late Stage**	1.62 (0.66-4.01)	0.29	-	-
**Tumor Methylation**	**0.88 (0.14-5.58)**	**0.89**	**0.91 (0.13-6.22)**	**0.92**
**Lymph Node Metastasis**	**6.12 (0.83-45.1)**	**0.075**	**5.48 (0.71-42.3)**	**0.098**
**Recurrence**	1.21 (0.19-7.64)	0.83	0.27 (0.02-0.56)	0.81

*OR, Odd ratio; CI, confidence interval.

*Multivariate model includes tumor methylation and lymph node metastasis only, to avoid model overfitting due to the limited sample size.
