# Supplementary material for: Integrated genome-wide DNA methylation and transcriptomic analysis prioritizes EPHA5 as a candidate non-invasive epigenetic biomarker for oral squamous cell carcinoma
Source: Front Oncol. 2026 Oct 2;16:1932336. doi: 10.3389/fonc.2026.1932336 (PMC13671974; doi:10.3389/fonc.2026.1932336)
Supplement: Supplementary file 3 [file Table6.pdf]

Supplementary Table S6. Univariate and multivariate logistic regression analyses of tumor methylation-associated expression loss in OSCC

**Parameters**	**Univariate Analysis**		**Multivariate Analysis***	
	**OR (95%CI)**	**p**	**OR (95%CI)**	**p**
**Age (per year)**	1.04 (0.95-1.14)	0.373	-	-
**Gender**	1.00 (0.13-7.45)	1.000	-	-
**Anatomical site**	1.58 (0.54-4.61)	0.410	-	-
**Smoking**	0.25 (0.03-1.86)	0.176	-	-
**Differentiation Degree**	0.55 (0.05-6.27)	0.630	-	-
**Tumor Stage (Early/Late)**	4.38 (0.41-47.02	0.223		
**Tumor methylation (continuous)**	**1.03 (0.98-1.09)**	0.225	**1.04 (0.98-1.11)**	0.172
**Lymph Node Metastasis**	1.50 (0.22-10.08)	0.677	2.40 (0.27-21.03)	0.428
**Salivary methylation (continuous)**	0.91 (0.74-1.13)	0.401	-	-

*OR, Odd ratio; CI, confidence interval.

*Multivariate model includes tumor methylation and lymph node metastasis only, to avoid model overfitting due to the limited sample size.
